# Comparative proteomics analysis of *Spodoptera frugiperda* cells during *Autographa californica* multiple nucleopolyhedrovirus infection

**DOI:** 10.1186/s12985-015-0346-9

**Published:** 2015-08-04

**Authors:** Qian Yu, Youhua Xiong, Hang Gao, Jianliang Liu, Zhiqiang Chen, Qin Wang, Dongling Wen

**Affiliations:** College of Food Science and Technology, Zhongkai University of Agriculture and Engineering, No. 501 Zhongkai Road, Haizhu District, Guangdong 510225 People’s Republic of China

**Keywords:** *Autographa californica* multiple nucleopolyhedrovirus, *Spodoptera frugiperda* cell, iTRAQ, Proteomics analysis, GO annotation, KEGG

## Abstract

**Background:**

Increasing evidence sugggest that in addition of balculovirus controling insect host, host cells also responds to balculovirus infection. However, compared to existing knowledge on virus gene, host cell responses are relatively poorly understood.

**Methods:**

In this study, *Spodoptera frugiperda* (Sf9) cells were infected with *Autographa californica* multiple nucleopolyhedrovirus (AcMNPV). The protein composition and protein changes of *Spodoptera frugiperda* (Sf9) cells of different infection stages were analysed by isobaric tag for relative and absolute quantification (iTRAQ) techniques.

**Results:**

A total of 4004 Sf9 proteins were identified by iTRAQ and 413 proteins were found as more than 1.5-fold changes in abundance. The 413 proteins were categorised according to GO classification for insects and were categorised into: biological process, molecular function and cellular component.

**Conclusions:**

The determination of the protein changes in infected Sf9 cells would help to better understanding of host cell responses and facilitate better design of this virus-host cell interaction in pest insect control and other related fields.

**Electronic supplementary material:**

The online version of this article (doi:10.1186/s12985-015-0346-9) contains supplementary material, which is available to authorized users.

## Background

Baculoviruses are a group of arthropod-specific viruses with rodshaped nucleocapsids of 30–60 nm × 250-300 nm, and they are a family of virus that is quite beneficial to humankind [1–3]. Baculovirus genomes consist of a circular double-stranded DNA (dsDNA) molecule of about 80–180 kb [4, 5]. There are several hundred baculoviruses that have been described in the literature which are highly specific for closely related groups of insects or crustaceans [6, 7]. Due to their restriction to host range and their ability to kill insects, baculoviruses have been used as microbial insecticides to control insect pests in agriculture and forestry [8, 9]. In the 1980s, they were developed as a powerful eukaryotic protein expression vector due to the extremly high expressions of viral gene in host cells [10].

Baculoviruses are classified into two genera, the nucleopolyhedroviruses (NPVs) and the granuloviruses (GVs); NPVs can be further subdivided into groups I and II based on phylogenetic studies of many genes [1]. Although there are many different baculovirus isolates, the best studied and the most widely used member of this family is *Autographa californica* Nucleopolyhedrovirus, a nuclear polyhedrosis virus (AcMNPV) [11]. AcMNPV is a large enveloped virus with a double-stranded circular DNA genome of about 130 kb. The complete sequence of AcMNPV has been determined [12]. AcMNPV infects a few insect lepidopteron species and it can be propagated easily in a cell line derived from the fall armyworm *Spodoptera frugiperda* (Sf), particularly the Sf9 clonal isolate [13].

The baculovirus infection cycle has been well studied. During the infection cycle, two progeny virion phenotypes, the budded virus (BV) and the occlusion-derived virus (ODV), are produced. The genotype of BV and ODV are identical, but each has characteristic structural components to accommodate its respective functions. The molecular life cycle is broadly divided into three consecutive phases according to gene expression programming [14]. In the early stage (0–6 h post-infection (hpi)), the nucleocapsids are transported through the nuclear membranes and migrate across the cytosol to the cell membrane. Genes requiring the transcriptional activity of the cell-encoded RNA polymerase II mainly act as master transactivators, which are essential for both subsequent viral gene expression and subversion of host cell activity by performing tasks common to other DNA viruses, including cell cycle arrest. Blocking apoptosis by the viral protein P35 is also an activity that is required to establish productive infections [15]. In the late stages of the infection (6–18 hpi), the viral DNA begins to replicate and the nucleocapsids within the nucleus are enveloped with a lipid bilayer that resembles the inner nuclear membrane. The transition from early to late phase is marked by the onset of replication and the activity of a virus-encoded, α–amanitin-resistant RNA polymerase. Concomitant with the onset of the very late phase (18 hpi-), host transcriptional activity is significantly decreased and most protein synthesis is shut off of by 24 hpi.

During the last decade, 68 baculovirus genomes including AcMNPV, the most widely used BV, have been sequenced, offering a wealth of information on the genetic diversity, gene sequences, gene content, genome organisation and phylogeny of baculovirus genomes [4, 12, 16, 17]. In comparison, the proteome of AcMNPV-susceptible cell lines have not been fully sequenced which is not helpful to understanding the responses of AcMNPV-susceptible cell lines during infection, since better understanding of the host cell responses would allow for the rational design of strategies for bioprocess optimization. Nuno Carinhas and colleagues obtained the comparative proteome quantitation of *S. frugiperda* cells during growth and early baculovirus infection (6 hpi) using stable isotope labelling by amino acids in cell culture [14].

In the present study, we set out to investigate the proteome changes in Sf9 cells infected with AcMNPV. In contrast to previous study by Nuno Carinhas et al. [14], this study centred on the differentially expressed proteins among Sf9 cells of mock, 6 hpi and 12 hpi which allows identification and measurements of thousands of proteins. The findings of these proteins improve our understanding of host-virus interactions and the host response upon virus infection, and hopefully, engender support in further studies regarding optimization of baculovirus application.

## Results

### Identification of differentially expressed proteins among mock, Sf9 cells at 6 hpi and Sf9 cells at 12 hpi

In the current study, Sf9 cells at resting, Sf9 infected with AcMNPV for 6 h and 12 h were collected for protein extraction, digestion and iTRAQ labelling to provide more information about proteome in AcMNPV infection. In this study, Sf9 cells at different phases of infection were collected to globally characterise the expression changes of Sf9 cell proteins associated with AcMNPV infection (Sf9 cells at resting, Sf9 cells at 6 hpi and Sf9 cells at 12 hpi) and a total of 4004 proteins were present in at least two of the three biological groups. Among the statistically significant proteins detected by ANOVA test (P < 0.05), the redundancies that changed less than 1.5-fold were discarded. Following this criterion, a total of 413 Sf9 cell proteins were found as being significantly differentially abundant under at least two repeated identifications and quantifications (P < 0.05) (Additional file 1: Table S1).

### GO annotation of the differentially abundant proteins

**Fig. 1:**
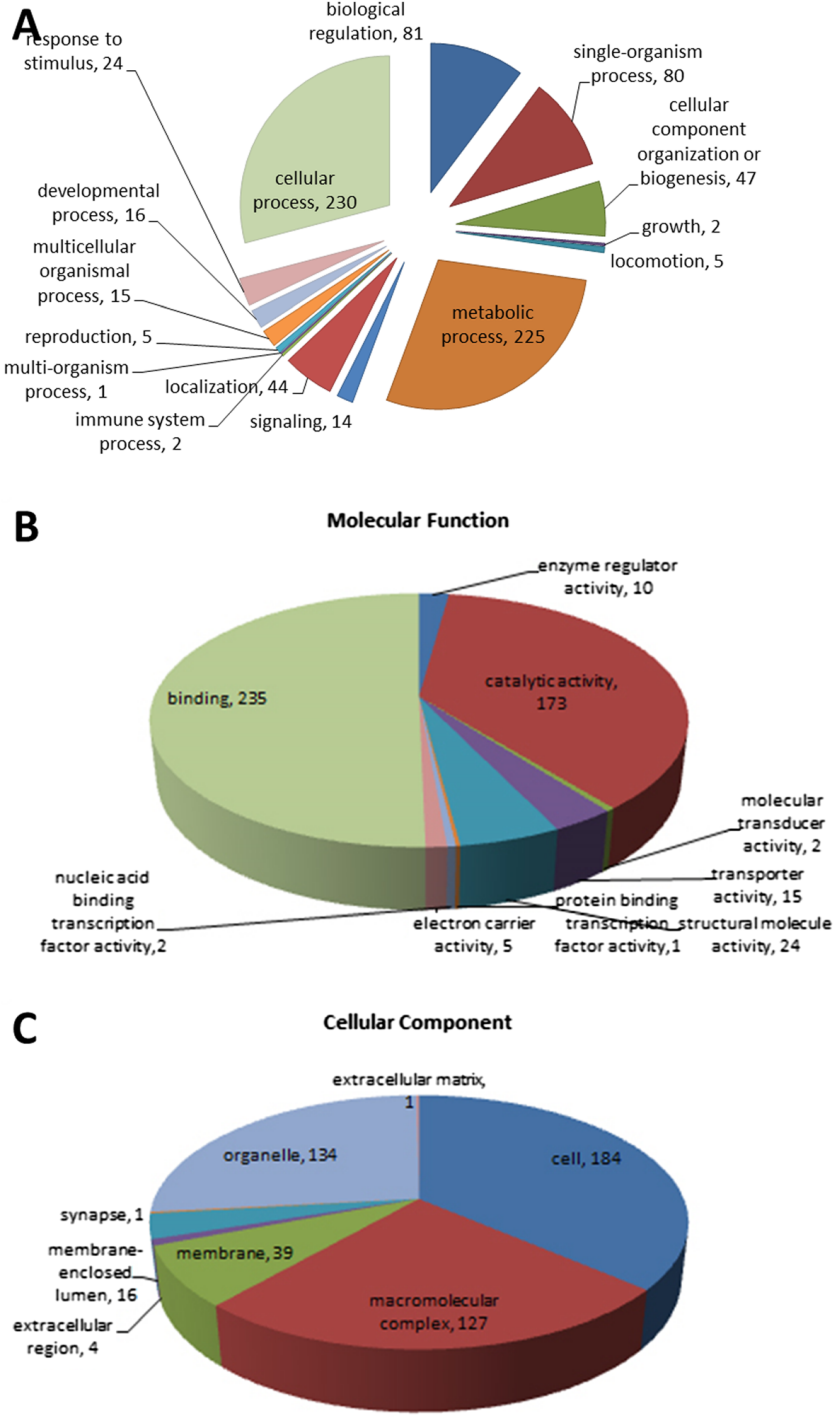
GO terms distribution. GO terms distribution in the molecular functions (**a**), cellular component (**b**), and biological process (**c**). In (**a**), binding and catalytic activity were the most represented biological processes. In (**b**), the most represented categories were cell, followed by organelle. In (**c**), the most represented categories were metabolic process and cellular process

The Blast2GO (Version 2.7.2) uses BLAST to find homologous sequences to input sequences and extract GO terms (Gene Ontology) for each obtained hit using existing annotations. These GO terms are assigned to the query sequence resulting in an assessment of the biological process, the molecular function and the cellular compartments represented. In this case, a GO category enrichment analysis was conducted using the 413 most abundant proteins to represent the overall trends of the specific functional categories that are enriched in Sf9 cells of different infection stages. Here, 292 proteins (70.70%) from Sf9 cells with 1156 annotation terms were linked to the GO cellular component, molecular function and biological process categories (Fig. 1).

The biological process category refers to a biological objective to which a gene contributes, but does not identify pathways. Examples of such processes include metabolism, cell communication, and sexual reproduction. In this case, the majority of proteins were found in cellular process (29.1 %), metabolic process (28.4 %), biological regulation (10.2 %) and single organism (10.1 %). Molecular function is defined as “what a gene product does at the biochemical level”. It is a very narrow definition, as it does not take into consideration the location of the event or the function in a broader, pathway or network context. In this category, most of the differential proteins were involved in binding (50.3 %), catalytic activity (37 %), structural molecule activity (5.1 %) and transport activity (3.2 %) (Fig. 1B). The binding functions were mainly at the intracellular level rather than external and included nucleotide and nucleic acid binding, protein binding and ion binding, while catalytic activities included transferase and hydrolase activities. The final category, cellular component, identifies where the gene products are found in the cell. These range from a general placement, such as in the “cell membrane” to more specific, such as the “histone deacteylase complex”. Here the differentially abundant proteins were mostly related to cellular components in the intracellular space (36.4 %), organelle (26.5 %), macromolecular complex (25.1 %), membrane (7.7 %) and membrane-enclosed lumen (3.2 %) (Fig. 1c).

### KAAS ----KEGG automatic annotation server

**Fig. 2:**
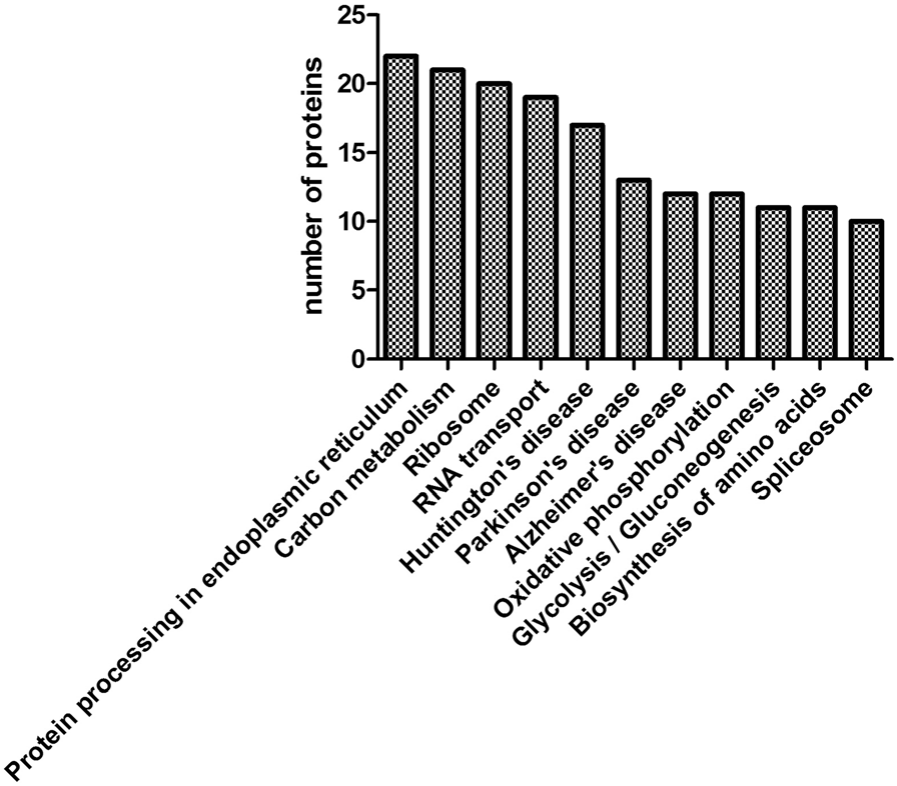
The most represented KEGG maps

The KEGG pathway is a collection of manually drawn pathway maps representing our knowledge on the molecular interaction and reaction networks. In this study, proteins of Sf9 cells at different infection stages were aligned with insect protein sequences in the KEGG GENES database using KAAS (KEGG Automatic Annotation Server) and annotated to biochemical pathways through the KO number of the homologous/similar proteins. We established pathway associations for 217 differential proteins with 207 unique KEGG orthologues. The most represented KEGG maps were protein processing in endoplasmic reticulum (22 members), carbon metabolism (21 members), ribosome (20 members), RNA transport (19 members), Huntington’s disease (17 members), Parkinson’s disease (13 members), Alzheimer’s disease (12 members), oxidative phosphorylation (12 members), glycolysis/gluconeogenesis (11 members), biosynthesis of amino acids (11 members) and the spliceosome (10 members) (Fig. 2). Several proteins/enzymes related to protein processing in the endoplasmic reticulum, carbon metabolism and ribosome were significantly up- or down-regulated after AcMNPV infection (Table 1).

**Table 1:** The significantly differentially expressed proteins in protein processing in endoplasmic reticulum, carbon metabolism and ribosome

Map Name (Map ID)	proteins	P value
protein processing in endoplasmic reticulum (ko04141)	Calreticulin	0.001566
	Protein disulfide-isomeras	0.021802
	Protein disulfide-isomerase A6	5.08E-05
	UV excision repair protein rad23	0.000256
	B-cell receptor-associated protein 31	2.97E-05
	UDP-glucose:glycoprotein glucosyltransferase	0.006069
	Prolactin regulatory binding-element protein	5.74E-05
	Dolichyl-diphosphooligosaccharide--protein glycosyltransferase subunit DAD1	0.000858
	Protein disulfide-isomerase	0.010141
	Phospholipase A-2-activating protein	1.70E-06
	Heat shock protein 90 beta	0.001462
	EIF2 alpha subunit	4.49E-06
Carbon (ko01200)	Putative enolase protein	7.75E-05
	Citrate synthase	5.44E-06
	Alcohol dehydrogenase	1.80E-05
	Ribosomal protein L12	0.000358
	Putative enoyl-CoA hydratase	1.29E-05
	Glucose-6-phosphate isomerase	0.009698
	Triosephosphate isomerase	0.00035
	Serine hydroxymethyltransferase	0.000124
	Succinyl-coa synthetase beta chain	0.000882
	Glyceraldehyde-3-phosphate dehydrogenase	0.000301
	Isocitrate dehydrogenase	0.022008
	Malic enzyme	2.51E-07
	Isocitrate dehydrogenase	0.001185
	Hydroxyacyl-coenzyme	0.000241
Ribosome (ko03010)	Ribosomal protein S17	0.013101
	Ribosomal protein L12	0.0001
	Ribosomal protein L30	0.009919
	Ribosomal protein L37	2.82E-05
	40S ribosomal protein S15	0.000307
	Ribosomal protein L36A	2.31E-06
	28S ribosomal protein S6	0.004214
	Ribosomal protein L1	0.000152
	Ribosomal protein S5 (Fragment)	0.000923
	Mitochondrial ribosomal protein S21	0.002409
	Ribosomal protein L6	3.78E-06
	Ribosomal protein S9	1.33E-05
	Ribosomal protein L28	1.55E-05

**Fig. 3:**
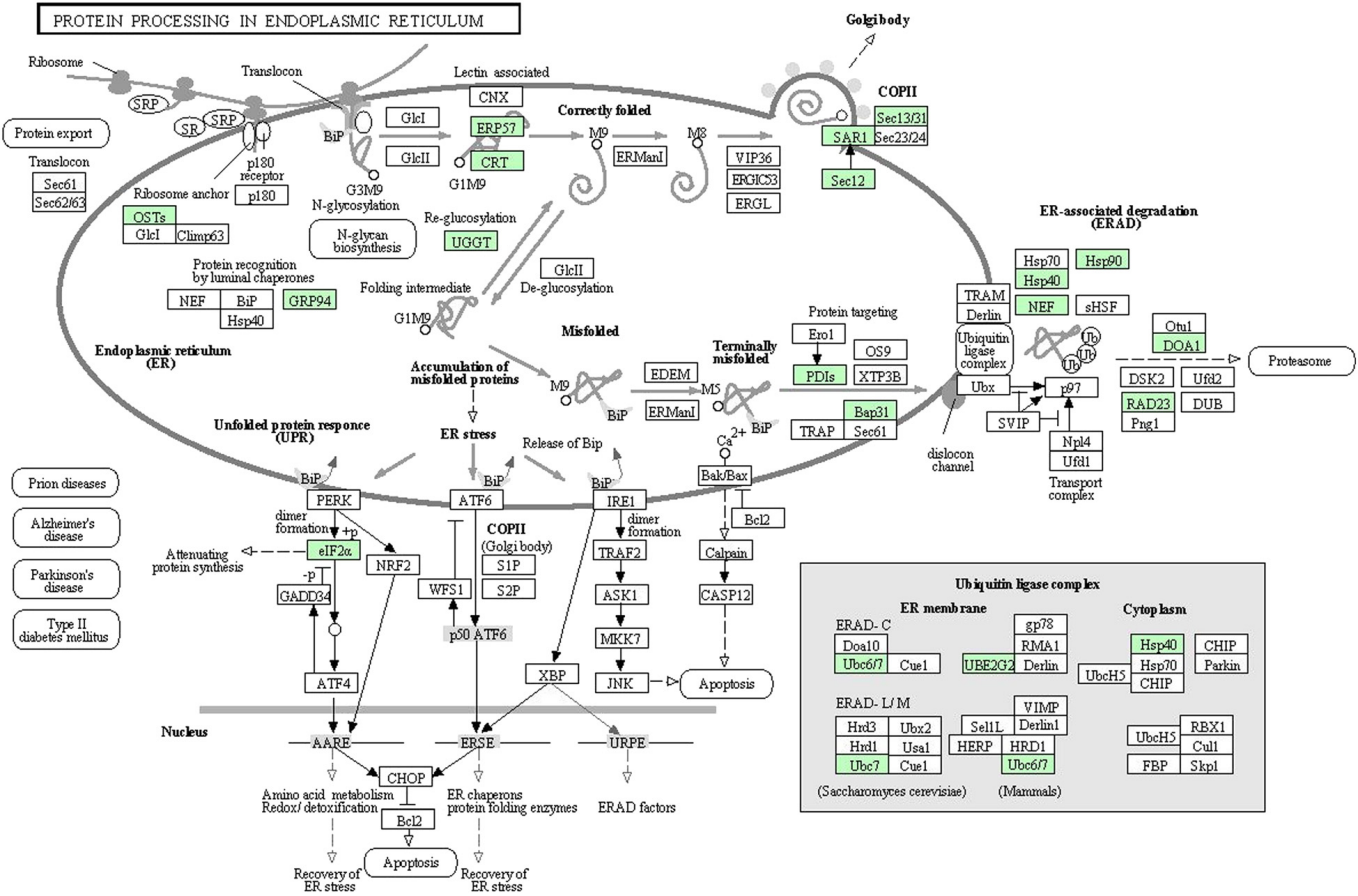
Representative KEGG pathway for protein processing in endoplasmic reticulum. The differentially expressed proteins were mapped as green

**Fig. 4:**
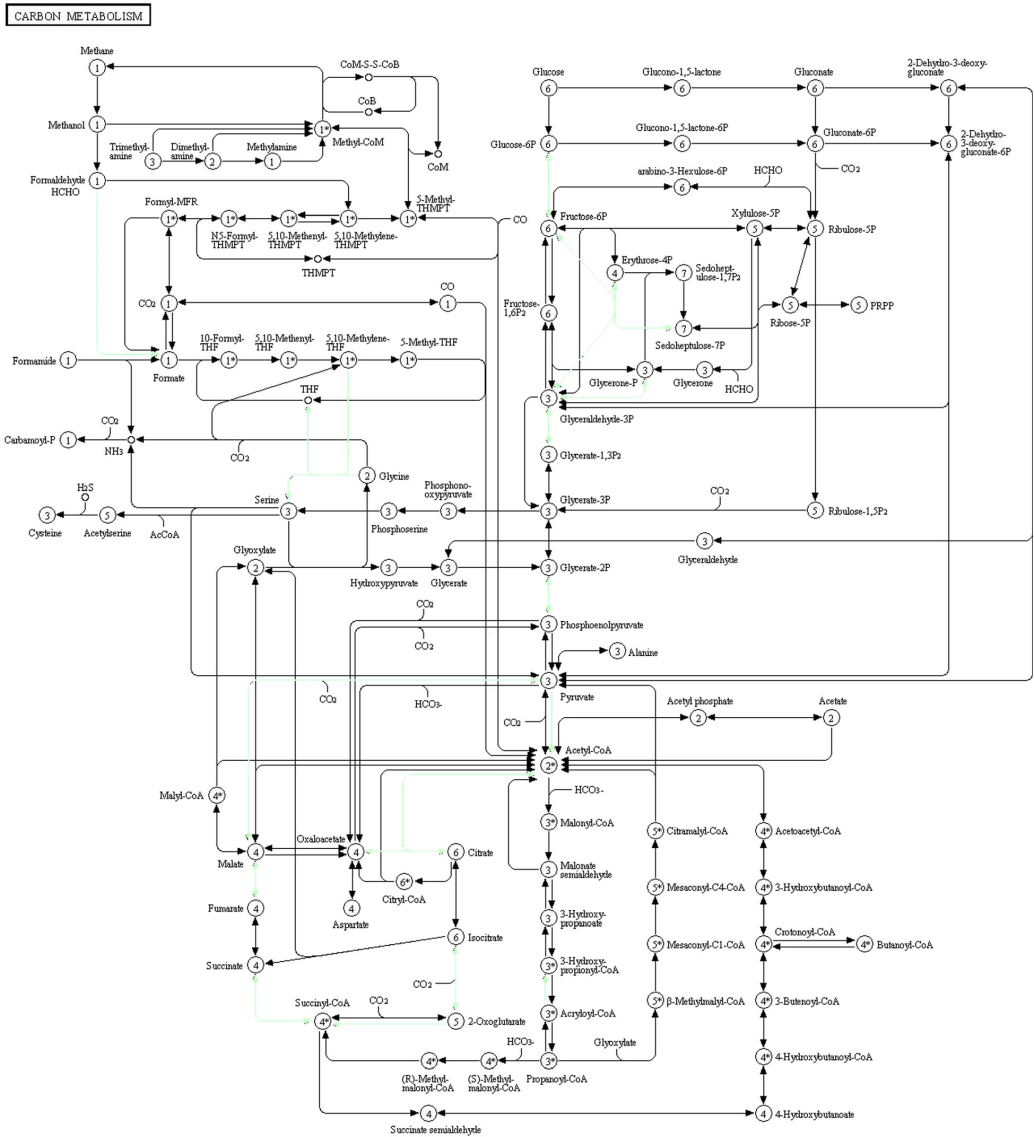
Representative KEGG pathway for carbon metabolism. The differentially expressed proteins were mapped as green

**Fig. 5:**
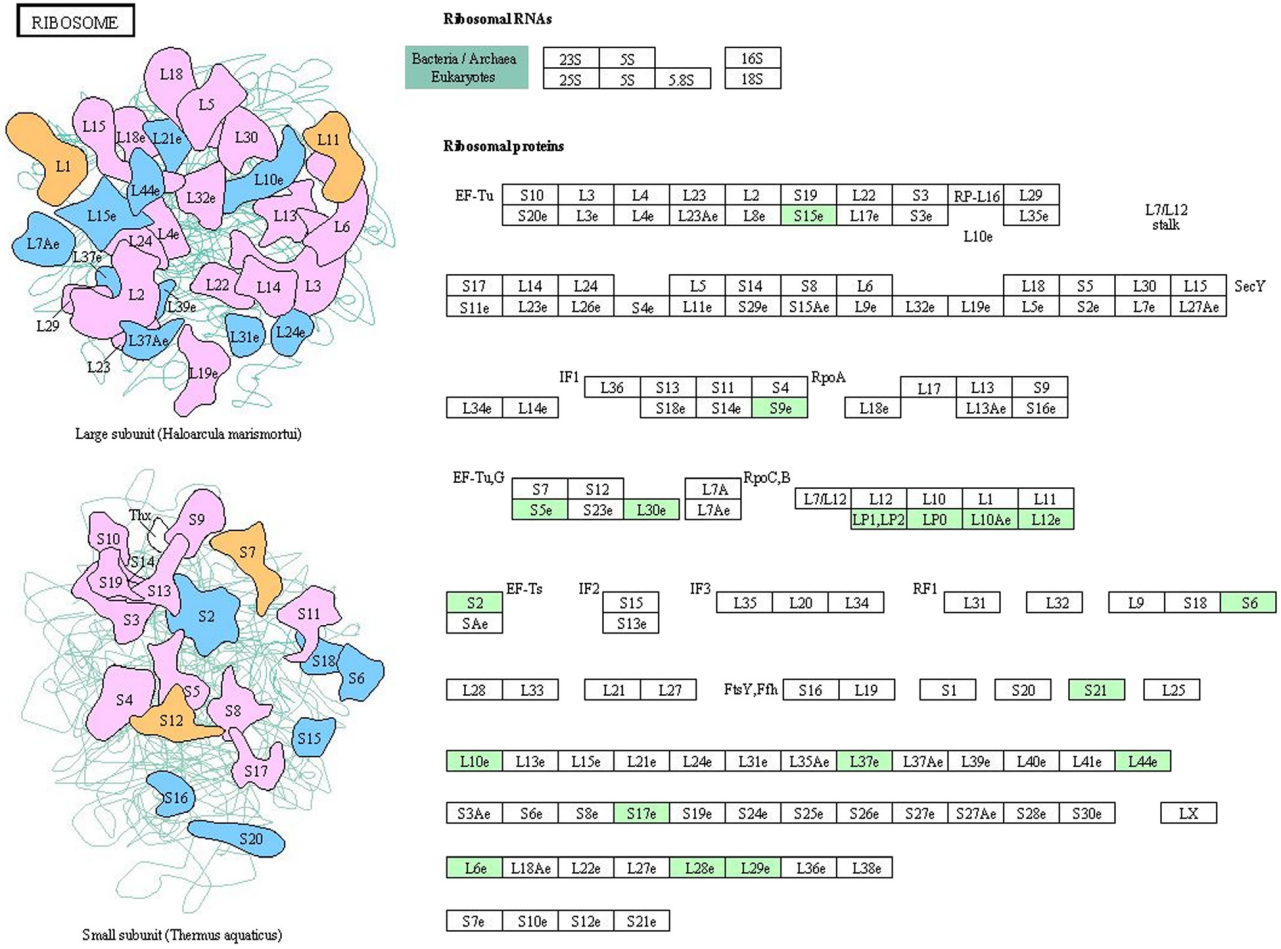
Representative KEGG pathway for ribosome. The differentially expressed proteins were mapped as green

For protein processing in the endoplasmic reticulum, AcMNPV is entirely dependent on the Sf9 cell translational machinery. We found that the expression of Calreticulin, EIF2α, Heat shock protein 90β and glycoprotein glucosyltransferase, among others, in Sf9 cells which are closely related to protein translation were significantly changed after AcMNPV infection. These proteins are involved in the translation of AcMNPV proteins using the Sf9 cell translation system. Concerning carbon metabolism, the gluconeogenesis/glycolysis pathways held the largest number of proteins. As no gene for an enzyme involved in energy metabolism has been found in any sequenced baculovirus, AcMNPV have to rely on Sf9 cell enzymes for energy metabolism (Fig. 5). The proteins with differential abundances involved in protein processing, carbon metabolism and ribosome were searched in KEGG (http://www.genome.jp/kegg/) and colored in Fig. 3, Fig. 4 and Fig. 5, respectively.

### Protein-protein interaction analysis

**Fig. 6:**
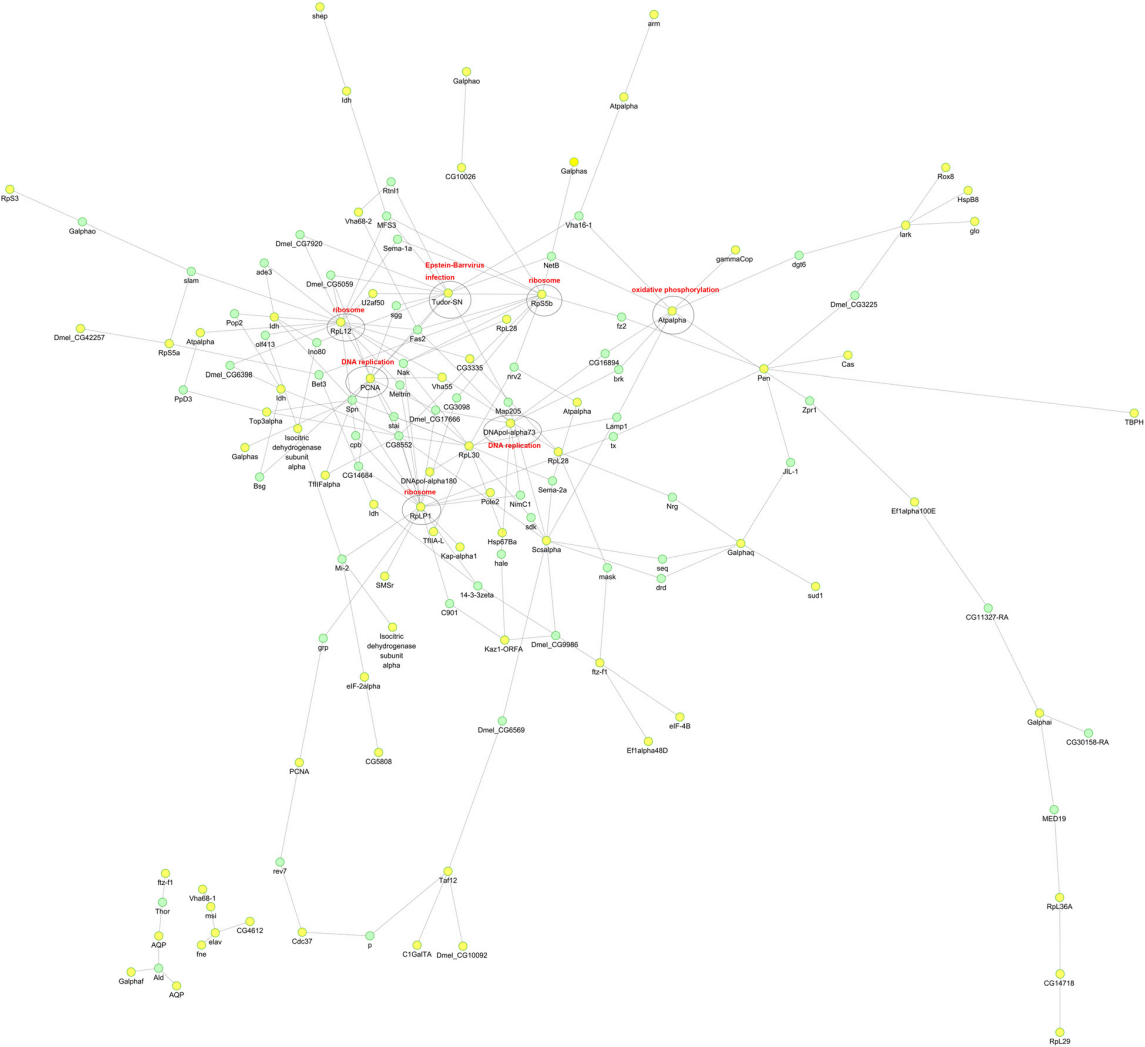
Protein-protein interaction networks. Network illustration of interactions among proteins from three groups of Sf9 cells. Yellow spots: significantly differentially expressed proteins. Green spots: proteins that did not expressed differentially

In addition to KEGG, we also constructed a protein-protein interaction network using STRING Database version 9.0. As shown in Fig. 6, the differentially expressed proteins were mainly enriched in the term “ribosome”, “DNA replication”, “Epstein-Barr virus infection” and “oxidation phosphorylation”. The ribosome and its related proteins were involved in translation, which can facilitate viral protein production. For host DNA replication, although cellular DNA replication is arrested, AcMNPV can utilize the host replication machinery to facilitate viral replication. The enrichment of “ribosome” and “DNA replication” were consistent with the high replication of AcMNPV in Sf9 cells.

## Discussion

Previous studies have accumulated rich knowledge about baculovirus gene expression and function during infection. These contain almost the entire whole process of infection, including virus entry, movement into the cell nucleus, DNA unpackaging, early transcription, DNA replication, late transcription, translation, budded virus (BV) assembly, BV budding, occlusion derived virus (ODV) assembly, occlusion, and release of occlusion bodies [18, 19]. However, unlike viruses such as AcMNPV, information on the gene expression and function in baculovirus-susceptible cells is very limited in the main public gene and protein databases. Salem et al. have investigated the impact of AcMNPV on host gene expression in cultured *S. frugiperda*, Sf21 cells, and found that the majority of the host genes were down-regulated over the time course of infection, although a small number were up-regulated [20]. Their study confirmed that host transcription was shut off. In addition, their study also provided a close estimate of the fold changes of the shut off for many genes. Since the direct cross-talk between virus and host occurs mainly at the protein level, the gene expression changes of the host cell could not reflect host cell responses accurately. Further study by Nuno Carinhas et al. demonstrated proteomic analysis of protein expression changes in Sf9 cells associated with culture growth and baculovirus infection. However, they focused on the transition from early to late phase (around 6 hpi) to investigate the establishment of infection and a total of 648 high-confidence hits were identified as protein homologues from 31 species and 2BV genomes [14]. It must be noted that the global changes of protein abundances in Sf9 cells at different infected stages (6 hpi and 12 hpi) were qualitatively and quantitatively analysed by iTRAQ in this study. This will help us understand the Sf9 cell response upon baculovirus infection and baculovirus infection pathways. Better understanding of the interaction between host cell and baculovirus would also help in optimizing the applicable area of baculovirus.

iTRAQ along with LC-MS is a robust protein discovery technique, allowing the determination of which proteins are affected in Sf9 cells during infection. Here, a total of 4004 Sf9 cell proteins were shown to be differentially expressed during AcMNPV infection. In fact, of these 4004 differentially expressed proteins, 413 were found to be significantly differentially abundant under at least two repeated identifications and quantifications, most of which are closely related to protein translation, protein processing and carbon metabolism.

In this study, 413 differentially expressed proteins were used to perform GO annotations. The functional annotation will help us to purposefully analyse the differentially expressed unigenes caused by virus infection. GO terms involved in cellular and metabolic process, binding, catalytic activity, cells and organelles were analysed by KEGG maps. Upon viral infeciton, a vast number of intracellular pathways in the host are activated/deactivated and profound metabolic changes occur. As a result, several effects arise, such as cellular cytoskeleton rearrangement,cell cycle arrest, apoptosis inhibition, metabolism subversion and host protein synthesis shut-off [21]. As far as cellular processes were concerned, genes involved in protein processes in the endoplasmic reticulum had regulated expression. Since the AcMNPV have no complex organelles, they are entirely dependent on the Sf9 cell translation machinery. Here, we showed that calreticulin, protein disulfide-isomerase A6, UV excision repair protein rad23, glycoprotein glucosyltransferase, prolactin regulatory binding-element protein, heat shock protein (HSP) 90 beta, and eIF2α, among others, were significantly differential expressed in Sf9 cells at different stages. Even actin, the typical control in eukaryotic cell experiments were significantly changed in AcMNPV infected cells. The significant changes of actin confirm host cellular cytoskeleton rearrangement upon infection. Calreticulin is a multi-functional calcium-binding chaperone that has several functions in protein folding, maturation, and trafficking [22, 23]. As an endoplasmic reticulum (ER)-resident chaperone, calreticulin keeps control of both exogenous and endogenous proteins [24]. These results contribute to the point that viruses rearrange the cytoskeleton not only through affecting actin expression, but may also by regulating other trafficking associated proteins to facilitate their own intracellular trasport. Previous studies have shown that translation factors, including eukaryotic initiation factors (eIF3-6, Eif1A, eIF3-2b) and elongation factor (EF1d) were commonly found up-regulated [25]; here, we found that eIF2 was also significantly expressed during the infection. AcMNPV also have to utilise Sf9 cell chaperones to facilitate the rapid synthesis of viral proteins. Our findings here about HSP90 expression are consistent with previous studies on Sf9 cell gene expression [20, 26]. The marked changes of HSP0 and other HSPs also suggested an ER hetereostasis upon infection. For glycoprotein glucosyltransferase and protein disulfide-isomerase A6, their differential expression may help with correct viral protein folding. Our studies here, together with previous studies that most host transcript levels were not significantly changed [27], suggested a post-translational regulation upon virus infection.

A number of proteins associated with the ribosome, the primary site of biological protein synthesis, were also differentially expressed throughout the AcMNPV infection, including ribosomal protein S17, S9, S5, S21, L1, L12, L30, L37, L6, and L28, as well as the 40S ribosomal protein S15 and the 28S ribosomal protein S6. Many ribosome proteins have been linked with cell structure, protein translation and protein biosynthesis [28, 29]. In uninfected cells, ribosomal proteins are typically expressed at a rate enough to maintain a constant ratio to rRNA under physiological conditions. It has been reported that in AcMNPV-infected Sf9 cells, a number of ribosomal proteins were dramatically changed. Our findings confirm the protein level changes of these proteins. The differential expressions of these proteins suggest changes in protein biosynthesis and the process of translating mRNA into protein during virus replication.

A number of genes involved in carbon metabolism appeared to be differentially expressed during AcMNPV infection, including citrate synthase, glucose-6 phosphate isomerase, glyceraldehyde-3-phosphate dehydrogenase, enoyl-CoA hydratase, alcohol dehydrogenase, triosephosphate isomerase, serine hydroxymethyltransferase, succinyl-coa synthetase β chain, isocitrate dehydrogenase, malic enzyme, and hydroxyacyl-coenzyme. These proteins or enzymes participate in the citrate cycle, the pentose phosphate pathway, glycolysis and gluconeogenesis, and pyruvate metabolism, along with glycine, serine and threonine metabolism to meet the high demands for ATP and the synthesis of cellular components during infection. Infection requires a high energy supply. Nevertheless, AcMNPV has no gene for energy-related enzymes; they have to manipulate Sf9 cells energy generation and metabolism pathways to foster their own replication. Furthermore, we also found that fatty aicd metabolism was dramatically changed upon infeciton, which was consistent with previous results [14]. Taken together, these results suggested that virus actively regulated host metabolic pathways that were essential for their replication.

In summary, AcMNPV infection of Sf9 cells leads to remodeling of host cell and large production of viral genes. During infection, expression of cell proteins involved in protein processing, ribosome, carbon metabolism and RNA transport et al. were found to be significantly changed (Additional file 2: Table S2). These differentially expressed proteins of infected Sf9 cells showed us how Sf9 cells responsed to virus infection. In this study, a battery of uncharacterised proteins was also discovered by biological information comparisons. However, even with the iTRAQ technology and bioinformatics protocols, these proteins could not be identified and their functions are still unknown. The changed proteins exerted crucial physiological effects during AcMNPV infection, and may help us in future researches aimed to optimize virus-host interaction, and in enlarging their application in different area.

## Conclusions

In conclusion, a proteomics approach based on iTRAQ was applied to determine the differentially expressed proteins of Sf9 cells during AcMNPV infection which allowing the identification of 4004 proteins. Among these, 413 different proteins were significantly changed. These significantly changed proteins were useful to offer new insights on the dynamic host responses to infecton. Moreover, the changed proteins may also be helpful in further applied studies aiming to improve baculovirus use as a biopesticide or a gene delivery vector.

## Materials and methods

### Cell culture and treatment

The Sf9 insect cell line, the clonal isolate 9 from IPLB-Sf21-AE cells derived from the fall armyworm *Spodoptera frugiperda* [30], was cultured at 27 °C in Grace’s medium (Invitrogen Life Technologies) supplemented with 10 % fetal bovine serum, penicillin (100 μg/ml), and streptomycin (30 μg/ml). AcMNPV was donated by Dr. Yang Kai (School of Life Sciences, Sun Yat-sen University, China). BV titers were determined by TCID50 (50 % tissue culture infective dose) end-point dilution assay using Sf9 cells [31]. For infection experiments, Sf9 cells were grown to the desired cell density and inoculated with AcMNPV at an MOI of 5 infectious particles per cell. Approximately 100 million cells were harvested either before infection or at 6 h and 12 h post-infection, and were seeded by centrifugation. Thus, cells were divided into three groups: mock: cells at resting, 6 hpi: Sf9 cells infected with AcMNPV and harvested at 6 h post-infection, and 12 hpi: Sf9 cells infected with AcMNPV and harvested at 12 h post-infection. Each group was repeated at least three times. Cell pellets were washed with ice-cold PBS, instantly frozen and stored at −85 °C until further analysis.

### Sample preparation

Sf9 cells from different groups were lysed in basic RIPA buffer containing 25 mM Tris (pH 7.6), 150 mM NaCl, 1 % NP-40, 1 % Na deoxycholate, 1 mM EDTA, and supplemented with 1 mM PMSF and 1 × complete protease inhibitor cocktail (Roche, Switzerland) as previously described [32]. Then, cell lysates were centrifuged and the protein content in the supernatant was quantified using BCA Protein Assay Kit (Thermo Scientific, Milford, MA). Equal amounts of protein (20 μg) were subjected to SDS-PAGE.

### Digestion and iTRAQ labelling

Digestion and iTRAQ Labelling were performed according to previously described methods [33]. Briefly, protein samples (400 μg) of each group diluted in 100 mM dithiotreitol solution were incubated in boiling water for 5 min, cooled to room temperature (RT) and diluted with 200 μL UA buffer (8 M Urea, 150 mM Tris–HCl pH 8.0). Then, samples were transferred onto a 10 kDa ultrafiltration filter for centrifugation at 14000 × g for 15 min, and washed again with UA buffer. Subsequently, 100 μL of iodoacetamide (IAA) solution (50 mM iodoacetamide in UA buffer) was added to the filter and mixed for 1 min at 600 rpm, followed by incubation for 30 min at room temperature in darkness and centrifugation at 14000 × g for 10 min. This step was repeated twice. Then, 100 μL dissolution buffer (Applied Biosystems, USA) was added to the filter and centrifuged at 14000 × g for 30 min, and repeated again. Finally, 40 μL of trypsin buffer (Promega, 5 μg trypsin in 40 μL dissolution buffer) were added and digested at 37 °C for 16–18 h. The filter unit was transferred to a new tube, rinsed and centrifuged at 14000 × g for 10min. Resulting filtrates were collected and the peptide content was analysed at 280 nm.

For peptide labelling, an 80 μg peptide mixture from each group was labelled with iTRAQ Reagent-8plex multiplex kit (AB SCIEX, Foster City, CA) according to the manufacturer’s instructions. Protein sample from mock was labeled with reagent 113. Samples from 6 hpi and 12 hpi were labelled with reagent 114 and reagent 115, respectively.

### Strong Cationic-exchange (SCX) chromatography separation

The peptide mixture was reconstituted and acidified with 2 mL of buffer A (10 mM KH_2_PO_4_ pH 3.0 and 25 % (v/v) acetonitrile) and loaded onto a Polysulfoethyl^TM^ (PolyLCInc, Maryland, USA) column (4.6 × 100 mm, 5 μm, 200 Å). The peptides were eluted at a flow rate of 1000 μL/min with a gradient of 0–10 % buffer B (solvent A with 500mM KCl) for 25 min, 10–20 % buffer B for 10 min, 20–45 % buffer B for 5 min, and 45–100 % buffer B for 13 min. The elution was monitored by absorbance at 214 nm, and fractions were collected every minute. The collected fractions (about 30 fractions) were finally combined into 5 pools and desalted on C_18_Cartridges (66872-U, Sigma).

### MS/MS analysis and quantification

For each fraction, 10 μL of solution was injected for nanoLC − MS/MS analysis using a Q Exactive MS (Thermo Finnigan) equipped with Easy nLC (ProxeonBiosystems, now Thermo Fisher Scientific). The chromatographic column was balanced with buffer A (0.1 % Formic acid). Five μg of the peptide mixture was loaded with an auto-sampler onto a Thermo scientific EASY column (2 cm*100 μm 5 μm-C18) and separated with the Thermo scientific EASY column (75 μm*100 mm 3 μm-C18) at a flow rate of 250 nL/min. A linear gradient of buffer B (84 % acetonitrile and 0.1 % Formic acid) used here was: a segmented gradient from 0–35 % (v/v) in 100 min, from 35–100 % (v/v) in 8 min, and then at 100 % (v/v) for 12 min.

The mass spectrometer data were analysed using the Q-Exactive mass spectrometer (Thermo Finnigan, USA) in the positive ion mode with a selected mass range of 300–1800 mass/charge (m/z). Resolving power for the Q-Exactive was set as 70000 at m/z 200 for the MS scan. Determination of the target value was based on predictive Automatic Gain Control (pAGC). Dynamic exclusion was 40.0 s. Survey scans were acquired at a resolution of 70000 at m/z 200, and resolution for HCD spectra was set to 17500 at m/z 200. Normalised collision energy was 30 eV and the under-fill ratio, which specifies the minimum percentage of the target value likely to be reached at maximum-fill time, was defined as 0.1 %. The instrument was run with peptide recognition mode enabled.

### Data analysis

The raw MS/MS spectra data were searched and identified using Mascot 2.2 and Proteome Discoverer1.4 (thermo). The database used in this study was UniProtKB 2014101053FC0RBNV8.fasta, released by Uniprot in August 2014. Assembling protein identifications were qualitatively analysed by Proteome Discoverer 1.4 software. All data were reported based on 99 % confidence for protein identification, as determined by the false discovery rate (FDR) ≤1 %. Statistical analysis was conducted using a one-way ANOVA. P-values ≤ 0.05 by Tukey's test were considered significant. Among the statistically significant proteins detected by the ANOVA test (p < 0.05), protein abundances that changed less than 1.5-fold were discarded.

### Bioinformatics

Proteins that were found to have a statistically significant difference in abundance among mock, Sf9 cells at 6 hpi and Sf9 cells at 12 hpi were then further analysed for functional and biological relevance. These proteins were classified by their gene function and also by biological pathways using the freely available gene ontology (GO) provided by the Gene Ontology Consortium. The retrieved sequences were locally searched against NCBI nr using the NCBI BLAST+ client software (ncbi-blast-2.2.28 + −win32.exe) to find homologous proteins from which the functional annotation was transferred to the targeted proteins. Here, the top 10 blast hits with an E-value less than 1*e*-3 for each query proteins were retrieved and loaded into Blast2GO (Version 2.7.2) for Gene Ontology (GO) mapping and annotation. In addition, differentially expressed proteins were sent to the Search Tool for the Retrieval of Interacting Genes/Proteins (STRING; http://string.embl.de/) to build a functional protein association network.

## Additional files

Additional file 1: Table S1. List of 413 proteins that had an individual P < 0.05 and changed not less than 1.5-fold in their relative abundance among Sf9 at mock, 6 hpi and 12 hpi. (PDF 390 kb) Additional file 2: Table S2. KEGG pathway. (PDF 187 kb)

## Abbreviations

AcMNPV: *Autographa californica* multiple nucleopolyhedrovirus
iTRAQ: isobaric tags for relative and absolute quantitation
GO: Gene Ontology
SCX: Strong Cationic-exchange.
