# Supplementary material for: Comparative proteomics analysis of Spodoptera frugiperda cells during Autographa californica multiple nucleopolyhedrovirus infection
Source: Virol J. 2015 Aug 4;12:115. doi: 10.1186/s12985-015-0346-9 (PMC4524103; doi:10.1186/s12985-015-0346-9)
Supplement: Additional file 1: Table S1. — List of 413 proteins that had an individual P < 0.05 and changed not less than 1.5-fold in their relative abundance among Sf9 at mock, 6 hpi and 12 hpi. (PDF 390 kb) [file 12985_2015_346_MOESM1_ESM.pdf]

Accession	Description	Proteins	Unique Peptides	MW [kDa]	calc. pI	ANOVA p value
K9J9U2	Actin depolymerization factor	4	3	16.9	7.2	6.36E-06
Q8WQJ2	60S acidic ribosomal protein P0	10	4	33.9	6.81	0.000193
H9IZ19	Uncharacterized protein	1	1	21.8	7.02	0.001081
E9NZS5	Arginine kinase	245	5	39.8	6.02	0.000276
E5CWM8	Elongation factor 1-alpha (Fragment)	1066	1	42.7	8.41	0.000245
F8TJD5	Elongation factor 1-alpha (Fragment)	939	1	42.7	8.24	0.001212
S4PMT5	Barrier-to-autointegration factor	2	2	10.1	5.48	0.0039
B3TEH3	Putative enolase protein (Fragment)	616	2	40.7	5.6	0.00035
G6DKM8	Mago nashi	2	1	17.2	6.52	0.000545
H2EVJ3	Elongation factor 1 alpha (Fragment)	8	2	16.1	6.67	0.00208
Q6F467	Ribosomal protein S17	6	1	15.3	9.7	0.001105
E3UJZ8	Calmodulin	5	9	16.8	4.22	3.10E-05
S4PXW2	Lamin C	1	2	70.2	6.89	0.023153
C7SZ24	Elongation factor 1-alpha (Fragment)	329	1	38.6	8.05	0.006246
E2EI06	Isocitrate dehydrogenase (Fragment)	1040	1	26.9	7.47	0.000258
Q86CZ0	ADP/ATP translocase	3	3	32.8	9.83	0.00026
B9U7K0	Elongation factor-1 alpha (Fragment)	19	2	29.1	8.22	0.000956
D1LYK5	Ribosomal protein L12	4	1	17.5	9.63	0.0001
I4DKA8	Tankyrase	3	1	12.7	5.27	0.002511
B7SET3	Elongation factor 1-alpha (Fragment)	751	1	45.1	8.34	4.71E-05
G3K8T0	Heat shock protein (Fragment)	107	1	37.4	5.15	0.001134
Q3V6C6	Heat shock protein 90	63	1	82.4	5.07	0.038981
Q8I6Y8	EIF2 alpha subunit	6	11	37	4.97	9.46E-06
H9IX91	Uncharacterized protein	1	1	41.7	6.74	0.021005
Q5UAT9	60S acidic ribosomal protein P2	4	1	11.5	4.74	0.044428
H9CV61	Elongation factor 1-alpha (Fragment)	766	1	45	8.24	0.013316
E3UPC7	Ribosomal protein L30	6	4	12.4	9.66	0.010668
Q1HPW4	Eukaryotic translation initiation factor 3 subunit I	2	2	36.9	6.11	0.00012
E3UUW3	Ribosomal protein L37	8	4	10.6	11.78	9.27E-06
A7UFP8	Elongation factor 1-alpha (Fragment)	755	1	44.7	8.48	0.012829
S4NRF9	40S ribosomal protein S15 (Fragment)	7	4	14.9	10.18	7.07E-05
E2EFQ3	Elongation factor 1 alpha (Fragment)	143	1	16.1	8.57	9.57E-05
Q8MYA5	Proliferating cell nuclear antigen	7	6	29	4.78	0.001033
Q1HPV7	Histone H2A	4	3	13.4	10.55	4.61E-08
S4NN37	Bicaudal (Fragment)	4	2	18.9	7.47	0.003817
A9XZA5	Putative pre-mRNA splicing factor RNA helicase (Fragment)	3	2	21.3	8.43	0.018548
E1U7I5	UDP-glucose pyrophosphorylase	3	2	57	7.88	0.000582
S4PGL2	Phosphopantothenoylcysteine	2	1	8.1	5.29	0.000151

	synthetase (Fragment)					
L7QLT0	Triosephosphate isomerase (Fragment)	91	1	16.2	5.33	0.008279
G6CMA9	Poly A binding protein	4	1	67.2	9.47	2.17E-05
S4PCX5	Ribonucleoside-diphosphate reductase small chain	2	1	43.6	5.44	5.87E-05
H9JMC0	Uncharacterized protein	1	1	42.4	8.05	0.000492
S4PS66	Tcp-1eta (Fragment)	2	2	35.8	7.43	1.06E-06
G6DQ61	Chaperonin	2	1	58.9	7.06	2.70E-05
G6CKL7	V-type proton ATPase subunit E	9	6	26	8.48	0.000196
I4DP84	Citrate synthase (Fragment)	1	1	32	6.52	1.69E-06
H9IT95	Uncharacterized protein	3	3	45.8	4.65	0.002483
H9J1E8	Proteasome subunit beta type	1	1	24.1	5.62	4.82E-05
I4DJK3	Protein kinase C inhibitor	2	1	13.9	6.8	0.005779
I4DJ87	ATP synthase delta chain, mitochondrial	3	3	17.1	6.58	5.11E-06
H9JCM2	Uncharacterized protein	2	2	51.7	6.57	0.025633
G6DNL4	Uncharacterized protein	2	1	55.9	6.02	0.016618
G6DH09	Mitochondrial carrier-like protein	1	3	33.1	8.95	0.012484
G0T406	Alcohol dehydrogenase	11	3	40	6.89	0.000138
L7R6U6	Putative enolase protein (Fragment)	26	1	40.6	6.04	0.022211
Q308N8	Glutathione S-transferase	4	2	24.5	8.63	0.002324
I4DMH6	Calreticulin	2	2	45.9	4.61	0.001566
E3UKL7	Myosin heavy chain (Fragment)	1	2	41.7	8.41	0.00116
Q5I7M6	Lark-PA	4	1	38.5	9.22	0.004344
H9JEB5	Uncharacterized protein	3	1	18.9	5.08	0.011133
E7DZ32	Ribosomal protein L36A	6	3	12.3	10.61	1.06E-05
I4DK67	Boca protein	4	3	21.6	4.89	0.003669
S4P2L5	Uncharacterized protein (Fragment)	3	1	7.8	10.13	0.001248
H9JV88	Uncharacterized protein	4	2	9.3	9.55	0.003566
C9S271	Putative enoyl-CoA hydratase	3	2	32	8.09	2.29E-05
M9UVR1	Voltage-dependent anion-selective channel	1	1	30.3	8.05	0.017154
D6BNT9	Glucose-6-phosphate isomerase	19	3	61.6	7.01	0.012198
H9J814	Uncharacterized protein	1	1	32.9	9.26	0.000356
D2SNQ8	Aldehyde dehydroxygenase (Fragment)	3	2	19.6	6.19	0.020096
B6VAH6	Proteasome subunit beta type	4	1	30.9	7.01	0.000205
G6DAS4	ATP synthase subunit epsilon, mitochondrial	1	1	10.4	10.39	0.000598
S4PCA3	Threonyl-tRNA synthetase	1	1	65.4	7.23	0.000172
I1E4Y7	Eukaryotic translation initiation factor 3 subunit G	3	2	30.3	8.34	3.98E-05

I4DIQ3	Protein disulfide-isomerase	5	2	55.3	4.74	0.002171
B2X122	Serpin-2	8	7	41.1	4.84	4.48E-05
Q95UN1	28S ribosomal protein S6	2	3	17.5	9.09	0.004214
S4PBN1	JmjC domain-containing histone demethylation protein 1 (Fragment)	3	1	8	10.17	0.000213
H9JA60	Uncharacterized protein	2	2	38.6	8.85	3.28E-05
G6CKB9	Uncharacterized protein	1	1	6.6	8.57	6.37E-05
E9KGW9	Glu-+ pro-tRNA synthetase (Fragment)	74	1	14.6	5.26	0.003585
Q1HPY3	Secreted protein acidic and rich in cysteine	5	4	37.4	4.97	0.000778
U5KC00	Aldo-keto reductase	7	2	35.8	6.8	0.000426
G6DME4	Acidic ribosomal protein	1	1	11.4	4.21	0.014593
E0D4V7	Phenylalanyl-tRNA synthetase beta subunit	1	2	65.2	6.49	0.005591
S4NQY4	N-acetyltransferase	4	2	13.7	6.79	0.009335
G6CIZ7	Triosephosphate isomerase	114	1	26.7	7.34	0.003249
D1LYQ1	Ribosomal protein L29	6	1	8.4	11.82	0.025087
I4DIL8	Elongation factor 1 gamma	2	4	48.4	6.05	0.005662
H9IX66	Uncharacterized protein	1	1	80.8	7.2	0.00076
C5I0W6	Annexin	2	2	36.3	5.78	0.016064
G6DIQ9	Tudor micrococcal nuclease	2	4	100.3	8.31	0.005069
I4DNK8	Courtless	2	1	18.7	4.96	0.021802
I4DNY2	Clathrin coat associated protein ap-50	1	1	33.2	9.07	0.000596
G6CTH9	ER lumen protein retaining receptor	5	2	24.5	8.82	0.045324
G6CTA2	Serine hydroxymethyltransferase	1	1	51.4	7.47	0.000634
H9IV80	Uncharacterized protein	1	2	78.9	6.77	0.001307
S4P8J8	Geminin	2	2	19.4	4.65	0.0018
S4NQB0	Putative arginyl-tRNA synthetase (Fragment)	1	1	8.7	4.86	0.000126
H9JD02	Uncharacterized protein	2	1	38.8	8.16	0.001152
D2SNS6	Aldehyde dehydrogenase (Fragment)	4	1	17.7	5.29	1.80E-06
G6D3R7	Cytidylate kinase	1	2	22.3	6.77	0.000278
G6D794	Vacuolar protein sorting-associated protein VTA1-like protein	2	2	29.3	4.81	0.00491
G6D689	U2 small nuclear ribonucleoprotein A	2	2	29.7	8.6	0.000439
H9JSN0	Uncharacterized protein	3	1	45.1	6.77	0.005918
G6CP89	Uncharacterized protein	2	1	9.2	7.42	0.003722
S4P5Y2	Liquid facets (Fragment)	3	2	22.6	8.85	0.029308
H9JCH9	Uncharacterized protein	3	2	19.8	9.38	0.000717
I4DQ01	Clathrin coat assembly protein orange	3	2	21.7	5.4	2.70E-05
S4P4S4	Uncharacterized protein (Fragment)	1	1	7.6	9.64	0.04038
H9JAP5	Uncharacterized protein	25	3	31.4	6.64	0.014906

S4PW23	Serine hydroxymethyltransferase	1	1	51.5	7.46	0.044258
B2DBK1	Karyopherin beta 3	2	2	121.9	4.83	0.011642
G6CPM9	Ribosomal protein S34	1	2	22.4	9.63	5.98E-07
H9IZJ3	Uncharacterized protein	1	1	44.8	7.66	0.015169
G6DT06	Uncharacterized protein	1	2	81.3	7.49	0.002995
G9FL14	DNA supercoiling factor	2	1	38	4.64	0.000953
H9JR08	Uncharacterized protein	3	1	68.9	6.95	0.000334
S4PN79	UDP-glucose 4-epimerase (Fragment)	1	1	10.6	7.59	0.000327
D9N4J4	Tudor staphylococcus/micrococcal nuclease	4	2	98.8	8.37	0.024713
G6D4D4	Putative membrane trafficking protein emp24/gp25/p24 family member	4	1	11.3	6.05	0.002893
H9J1U7	DNA-directed RNA polymerase	7	1	205.9	7.97	0.000146
G6CVH5	Aspartyl-tRNA synthetase	2	1	37	6.62	2.38E-05
S4PBL3	Polypeptide N-acetylgalactosaminyltransferase 5 (Fragment)	1	1	31.5	7.18	0.046104
S4P7Q9	Myotubularin-related protein 2 (Fragment)	2	1	54.6	9	0.001038
V9VJY0	R2D2	1	2	36.7	6.2	1.47E-05
S4P487	Uncharacterized protein (Fragment)	1	1	10.8	8.87	1.94E-05
G6DQA4	Myosin vi	5	6	139.9	8.76	0.000523
G6DI16	Coatomer protein complex subunit alpha	1	1	67.7	8.59	0.049657
G6CX60	Putative Huntingtin interacting protein K	3	1	13.4	4.92	4.73E-05
G6DAQ7	Uncharacterized protein	2	1	15.5	8.97	0.000129
S4PS76	Glycine N-methyltransferase (Fragment)	2	1	23.4	5.64	0.001043
G6CIK4	Putative mitochondrial processing peptidase alpha subunit	1	1	10.2	10.35	0.002135
I4DNH5	Protein disulfide-isomerase A6 (Fragment)	4	2	35.6	8.75	5.08E-05
I4DNI3	Similar to CG1969	4	1	11.7	7.08	0.001928
S4PRZ2	Glia maturation factor beta (Fragment)	3	1	15.2	5.17	2.78E-06
H9J2S8	Uncharacterized protein	2	2	45.1	9.33	1.92E-06
R4ITA8	CG1785-like protein	1	2	53.5	9.98	0.008707
D3GDM2	Antennal esterase CXE11	1	1	60.1	5.88	0.00068
G6CKL9	HSP90 cochaperone CDC37-like proteinue	1	2	43.2	5.26	0.004208
H9J752	Uncharacterized protein	1	1	29	5.16	0.009956
H9JX14	Uncharacterized protein	1	1	17.8	5.43	0.000398

S4P8C3	Cbl-d (Fragment)	3	1	17.8	5.52	0.000223
I4DMW4	UV excision repair protein rad23	9	3	36.2	4.54	0.000256
H9J051	Uncharacterized protein	6	2	22.9	8.63	0.000215
G6DIH8	Clustered mitochondria protein homolog	4	9	177.8	7.05	0.005048
H9JHI6	Uncharacterized protein	1	1	17.1	5.92	0.001007
H9JQJ7	Uncharacterized protein	2	1	16.4	7.3	0.000219
H9JFY6	Uncharacterized protein	2	2	40.4	7.12	0.001968
Q1HQB5	S-adenosylmethionine synthase	4	2	44.5	6.4	0.000693
G6D354	THO complex subunit 2	1	1	149.6	8.07	0.000353
H9JH77	Uncharacterized protein	3	2	49.4	5.29	0.007221
D2SNL0	Oxidoreductase (Fragment)	3	2	31.3	6.11	0.0007
S4PB07	Nucleoporin NUP85 (Fragment)	1	1	13.3	8.51	0.047205
S4NZV0	Metalloprotease (Fragment)	1	1	12.2	6.8	3.59E-06
H9ISH7	Uncharacterized protein	2	1	116.9	8.7	0.023513
J9XNW6	Autophagy related protein Atg4-like protein (Fragment)	1	2	41.4	4.98	3.01E-05
H9JDY4	Uncharacterized protein	3	1	30.3	11.55	2.03E-07
G6CQB4	Phosphatidylinositol glycan	3	2	39.7	7.83	0.001312
G6DT55	Uncharacterized protein	1	1	18.3	4.65	0.03057
G6CX56	Uncharacterized protein	1	1	32.4	4.41	6.10E-05
G6CJU9	Mitochondrial aldehyde dehydrogenase	3	1	14.9	8.62	0.010131
G6D7A4	Uncharacterized protein	1	1	43.7	7.33	1.67E-05
G6D8L6	Mitochondrial import inner membrane translocase subunit TIM50-C	2	2	38.1	6.6	0.001188
S4P7F7	Protein FAM98A (Fragment)	2	1	19.4	5.06	0.000644
S4PEA4	Suppressor of G2 allele of SKP1-like protein (Fragment)	3	1	19.7	5.22	1.06E-05
G6DKG9	UDP-glucosyltransferase protein 3	1	1	14.2	10.33	5.77E-06
H9JQK7	Uncharacterized protein	1	1	40	7.58	0.012592
H9JEI1	Uncharacterized protein	2	2	72.7	7.42	0.000261
S4PE88	Nicotinate phosphoribosyltransferase (Fragment)	1	1	20.7	6.52	0.000276
I4DNR5	Transcription factor TFIIFbeta	3	1	18.4	9.31	0.001426
H9J2E4	Uncharacterized protein	6	1	17.2	8.48	3.26E-05
G6DP49	Uncharacterized protein	2	2	131.6	6.73	4.65E-06
G6CV32	Putative phosphatase 2C beta	1	1	42.3	4.89	0.000577
H9JBG4	Uncharacterized protein	1	1	16.5	7.01	0.000255
H9IT11	Uncharacterized protein	3	1	146.8	6.3	0.000596
G6DLM7	Uncharacterized protein	1	1	31.7	8.57	5.44E-07
H9JU60	Uncharacterized protein	3	2	58.9	8.27	5.45E-05

S4P621	Programmed cell death 6-interacting protein (Fragment)	1	1	20	7.37	0.001152
Q1HQ07	1110059p08rik-like protein	1	2	34.3	5.39	5.73E-05
G6D1A4	Putative WD repeat protein 26	1	2	61.9	6.74	0.000973
G6DDK6	Uncharacterized protein	2	1	23.7	6.44	0.004437
S4NN06	Hsc70-interacting protein 1 (Fragment)	1	1	32.8	4.94	0.001576
G6DPV5	Putative Tetratricopeptide repeat protein 1	2	1	21.3	4.73	0.00073
G6D6P8	Uncharacterized protein	3	1	53.7	9.14	0.033124
H9JRQ9	Uncharacterized protein	3	2	37.8	8.62	0.005669
H9ITA0	Uncharacterized protein	2	2	45.4	5.54	0.002746
H9IY28	Uncharacterized protein	1	1	77	7.18	0.004539
G6DFX7	Putative isoleucyl tRNA synthetase	2	1	138.2	7.56	9.97E-05
M9T0T9	ATP synthase subunit a (Fragment)	116	1	18.1	9.32	0.002101
B0LL83	Pyruvate dehydrogenase kinase	2	1	46.9	7.08	0.002149
H9JJ62	Uncharacterized protein	3	2	79.8	5.73	0.000218
G6CX02	Protein BCCIP homolog	2	1	32.1	4.44	2.35E-06
G6D5V7	Adenylyl cyclase-associated protein	1	1	57.6	6.11	0.001329
G6DQ30	Transcriptional regulator ATRX	6	2	224.9	7.25	0.006427
B2DBH5	Putative uncharacterized protein	1	1	29.6	4.36	0.023516
S4PW98	Smad nuclear interacting protein 1 (Fragment)	3	1	27.9	9.41	0.000554
H9JLI9	Uncharacterized protein	2	1	20.6	5.48	1.25E-05
S4PTH3	B-cell receptor-associated protein 31	1	1	25.3	7.94	2.97E-05
H9IU37	Uncharacterized protein	1	1	62.9	6.3	1.61E-05
S4PCM2	Interferon regulatory factor 2-binding protein 2-A	2	1	45.3	8.54	0.001478
G6CRM2	Uncharacterized protein	1	1	25.1	8.24	0.001057
H9J7D4	Uncharacterized protein	1	1	49.4	8.6	0.000299
G6DG28	Uncharacterized protein	3	1	51.5	8.92	0.002161
Q3V5Y5	Carotenoid-binding protein (Fragment)	8	1	23.3	5.5	0.000539
G6DMD9	Uncharacterized protein	1	1	21.8	9.13	0.000141
Q8T9W9	cGMP-dependent protein kinase	11	3	83.9	6.06	3.46E-06
H9J9X5	Uncharacterized protein	2	1	22.7	4.73	0.003455
S4PEX9	DNA polymerase	5	2	67.2	8.76	0.009825
E9LFS8	Cytochrome c oxidase subunit 1 (Fragment)	1	1	22.6	4.98	1.75E-05
I4DNC1	Cytochrome C1 (Fragment)	5	1	33	8.05	1.38E-05
S4NNL1	UDP-glucose:glycoprotein glucosyltransferase	3	1	88.8	6.07	0.006069
G6DK55	Uncharacterized protein	3	1	54.7	5.92	0.000625

H9JU86	Uncharacterized protein	2	1	28.1	7.25	9.16E-05
H9JK13	Uncharacterized protein	1	1	25.6	8.5	0.000427
G6D8L4	Sorbitol dehydrogenase	3	1	28.7	8.18	0.04319
Q1HPT3	Heterogeneous nuclear ribonucleoprotein A1	1	1	35	7.06	0.042593
G6CNY0	Uncharacterized protein	2	1	103.1	7.27	0.005758
H9JQH9	Uncharacterized protein	2	1	26.2	8.1	0.00152
O77129	Period (Fragment)	1	1	37.7	5.95	2.82E-05
S4PL34	Nuclear RNA export factor 1	3	1	50.8	7.21	0.005581
G6D2U8	Uncharacterized protein	10	1	26.2	10.43	0.020337
H9J5V8	Uncharacterized protein	2	1	88.1	8.94	0.01787
H9JCQ2	Uncharacterized protein	1	1	36.4	8.98	1.19E-05
G6D3G4	Uncharacterized protein	1	2	115.8	5.86	2.85E-05
H9JRV8	Uncharacterized protein	2	1	35.6	5.78	2.98E-05
S4NSX0	Pre-mRNA-splicing factor RBM22	3	1	45.6	8.59	0.016475
H9JFV2	Uncharacterized protein	1	1	118.9	7.06	0.001079
H9J214	Uncharacterized protein	2	1	33.5	9.47	1.67E-06
H9JEG2	Uncharacterized protein (Fragment)	2	1	124.7	7.03	0.000979
G6CIU6	Uncharacterized protein	2	1	184.4	6.34	0.00911
H9JPT1	Uncharacterized protein	1	1	49	8.06	0.026888
S4PX05	Sortilin-related receptor (Fragment)	1	1	44.8	5.88	0.003655
G6CSA9	Ultrabithorax binding protein 1	1	1	44.9	8.35	0.048667
G6DGI3	Putative WD-repeat protein	2	1	46.2	8.68	0.000761
H9JU67	Uncharacterized protein	1	1	145.3	8.53	0.000462
I4DJM3	Metalloendopeptidase	2	1	47.4	6.93	0.003814
G6CUY8	Uncharacterized protein	1	1	44.3	4.77	0.003793
S4NNP8	Scraps (Fragment)	3	1	44	6.1	0.002888
G6DP16	Uncharacterized protein	1	1	92.8	5.3	0.000207
G6DBR6	Prolactin regulatory binding-element protein	4	1	45.5	8.56	5.74E-05
G6CMI3	Uncharacterized protein	1	1	42.5	8.19	0.00046
H9JUP2	Uncharacterized protein	3	1	42.1	5.76	0.000623
G6DCM1	Uncharacterized protein	1	1	56	5.26	6.54E-07
G6DRI3	Uncharacterized protein	4	3	200.4	5.12	0.000344
I4DNR0	Uncharacterized protein	4	1	43.4	4.94	0.000243
H9JFU5	Uncharacterized protein	2	1	64	7.93	0.002013
H9JDN4	Uncharacterized protein	1	1	54.3	7.17	1.50E-05
G6D0X1	Putative ebna2 binding protein P100	1	3	250.8	5.08	0.008573
A5JPL9	MSL3 protein	4	1	62.4	5.5	0.029685
H9JQY1	Uncharacterized protein	1	1	53.6	7.74	0.000482
S4NSU9	Succinyl-coa synthetase beta chain	1	1	49.8	6.81	0.002049
V9VJY3	Dicer 1 (Fragment)	1	1	153.9	5.26	0.000123
Q53EK0	Prolyl 4-hydroxylase alpha subunit	1	1	63.7	6.3	3.62E-05

G6DB46	Midasin	2	1	587.8	5.39	3.11E-05
H9JSF9	Uncharacterized protein	1	1	129.1	5.76	3.93E-05
G6DDK9	Uncharacterized protein	1	1	70.5	10.32	0.000343
H9J8V4	Uncharacterized protein	3	1	75.9	5.19	0.005108
H9IX25	Uncharacterized protein	2	1	74.1	8.07	0.033602
G6CYN2	Uncharacterized protein	2	1	76	7.83	4.06E-05
G6DGG9	Endonuclease-reverse transcriptase	1	1	82.5	9.73	0.038186
G6CNG9	Cysteinyl-tRNA synthetase	1	1	83.9	6.48	1.29E-05
G6DFF9	Phosphoglucomutase	1	1	68.1	5.48	0.000157
G6CUR1	Hemicentin-1	1	1	71.8	7.4	0.000407
H9J651	Uncharacterized protein	1	1	75.8	9.33	6.97E-05
H9JLR9	Uncharacterized protein	4	10	1538.9	5.08	2.94E-05
H9ISY0	Uncharacterized protein	2	1	109	8.31	0.000173
H9JIE7	Uncharacterized protein	2	1	149.3	6.98	6.52E-05
D0VY44	Neuroglian	1	1	139.9	6.11	0.024891
G6D6E9	DNA polymerase	1	1	187	6.28	0.017575
D9D6W7	Elongation factor-1 alpha (Fragment)	45	1	16.9	8.02	0.003124
V5LFB1	Elongation factor 1 alpha (Fragment)	107	1	20.3	7.91	2.11E-06
F0UYY3	Thymosin isoform 2	6	1	14.7	5.05	3.51E-06
E9LFT9	Elongation factor-1 alpha (Fragment)	244	1	18	8.28	8.43E-05
E2EG74	Elongation factor 1 alpha (Fragment)	244	1	18	8.29	0.001862
H9JJE6	Histone H4	4	1	11.4	11.36	2.34E-07
Q5QE34	Elongation factor 1-alpha (Fragment)	401	1	37.8	7.49	0.016148
E2EG63	Elongation factor 1 alpha (Fragment)	33	1	18	8.29	0.00048
O44308	Elongation factor 1-alpha (Fragment)	1878	1	45.1	8.48	7.32E-07
D4P236	Elongation factor 1 alpha (Fragment)	30	1	28	7.03	0.003385
H9JFB9	Uncharacterized protein	1	1	5.4	8	0.014269
E2EG88	Elongation factor 1 alpha (Fragment)	4	1	16.7	8.29	0.022567
C7E8S9	Elongation factor 1-alpha (Fragment)	612	1	42.7	8.41	0.028096
D4P290	Elongation factor 1 alpha (Fragment)	178	2	26.3	6.4	0.015201
H9JY79	Histone H2A	1	2	7.7	9.52	0.00017
D1LAB2	Elongation factor 1-alpha (Fragment)	604	1	45	8.34	0.03837
E9LFU9	Elongation factor-1 alpha (Fragment)	7	2	25.4	8.51	0.041823
G3K8R2	Heat shock protein (Fragment)	108	2	37.3	5.15	0.004125
H9JMB5	Uncharacterized protein	1	1	10.1	5.48	0.006066
H9JX71	Uncharacterized protein	3	2	47.2	6.48	0.000431
G6CL94	Uncharacterized protein	21	1	28.2	5.68	0.000124
I4DRR2	Splicing factor pTSR1 (Fragment)	1	1	25.6	8.24	0.000166
S4PIE3	Neuropathy target esterase sws (Fragment)	1	1	9.8	4.88	0.000223
Q5EPW0	Elongation factor 1 alpha (Fragment)	125	2	38.5	8.05	0.022192
G6DAB3	Ribosomal protein L1	14	1	28.6	9.82	0.000152

D3X5P2	Ribosomal protein S5 (Fragment)	46	1	21.1	9.57	0.000923
H9IZ08	Uncharacterized protein	1	3	42.1	7.44	0.000788
H9J567	Proteasome subunit alpha type	3	1	27.1	6.9	0.001203
F8UEY1	Glyceraldehyde-3-phosphate dehydrogenase (Fragment)	378	1	22	7.37	0.020499
S4NL80	Pleckstrin-like domain-containing family F member 2-like protein (Fragment)	1	1	9	10.01	6.18E-05
E2IV54	V ATPase A (Fragment)	1	2	33.2	5.52	0.00017
H9IUJ6	Transaldolase	3	1	37.1	7.2	0.013105
S4PMM4	Transgelin	4	1	20.9	7.75	6.52E-06
H9J1K4	Uncharacterized protein (Fragment)	4	2	12.7	9.95	0.03715
S4PXM6	DNA replication licensing factor Mcm6 (Fragment)	1	1	56	6.86	0.006464
H9JBL2	Isocitrate dehydrogenase [NADP]	134	1	46.1	6.7	4.55E-07
S4PSE3	Lark (Fragment)	1	1	32.5	8.88	0.00609
Q2F5S1	Microtubule-associated protein RP/EB family member 3	4	1	30.5	5.71	5.97E-05
H9JMJ8	Uncharacterized protein	2	1	19	9.13	0.000767
Q8ISR4	QM protein (Fragment)	1	1	6.3	9.76	0.038105
S4NTC3	eIF5B	2	1	69.5	8.25	1.26E-05
Q1HQC0	Mitochondrial ribosomal protein S21	3	2	11.7	10.07	0.002409
S4PD63	Diadenosine tetraphosphatase	3	2	16.1	5.52	0.005135
H9JFI7	Uncharacterized protein	2	1	38.2	8.97	0.030142
I4DL89	Coiled-coil domain-containing protein 72 homolog	1	1	7	10.05	0.001843
Q1HPQ3	Transcription initiation factor IIA subunit 2	2	1	12.5	8.81	0.000533
G6D1T2	Heat shock protein 70	1	1	82.7	9.31	5.86E-06
S4PT88	Dodo OS=Pararge aegeria PE=4 SV=1 - [S4PT88_9NEOP]	2	1	17.8	7.99	7.87E-05
S4PI06	Ubiquitin-specific protease (Fragment)	2	1	8.2	10.01	0.028797
G6DH38	Uncharacterized protein	3	2	19.4	8.59	4.61E-05
G6CY74	Malic enzyme	3	7	63.3	6.7	2.55E-05
H9J5Q7	Uncharacterized protein	1	4	34.7	5	0.001908
G6CTV3	Isocitrate dehydrogenase	3	1	42.5	7.72	3.32E-05
S4NNK0	Spectrin beta chain (Fragment)	1	3	148.2	5.07	2.64E-05
G6DL52	Putative DNA-directed RNA polymerase II 13.3 kDa polypeptide	4	2	13.5	5.83	1.85E-06
I4DJD2	Eclair	3	2	25	7.18	0.008024
I4DMG3	Ribosomal protein L6	7	2	30.7	10.92	3.78E-06
S4P7Y9	Putative leucine-rich	1	1	7.7	9.16	0.003021

	repeat-containing protein 47-like protein (Fragment)					
G6CQI5	V-type proton ATPase subunit G	7	1	8.9	5.1	2.72E-05
S4PGF1	Cullin-associated NEDD8-dissociated protein (Fragment)	1	1	16.3	7.84	0.000111
G6CWF2	Putative nuclear inhibitor of protein phosphatase-1	1	2	38.6	7.93	0.005303
S4P4F1	NMDA receptor-regulated protein (Fragment)	1	1	9.3	6.04	1.87E-05
S4NXV8	Guanine nucleotide-binding protein-like 3-like protein (Fragment)	1	2	35.8	9.45	0.039169
B2DBN5	Nucleosome assembly protein	2	1	44.7	4.81	0.043105
H9J2D4	Uncharacterized protein	1	1	44.2	6.58	0.00181
I4DID4	Ribosomal protein L6	18	1	30.7	10.73	3.50E-06
H9JB76	Peptidyl-prolyl cis-trans isomerase	4	2	20.3	7.27	0.002662
G6CX25	Coatomer protein complex subunit delta	1	1	62.8	8.44	3.18E-06
H9JY38	Galectin	3	2	24.3	6.77	0.000488
L7R0E7	GTP-binding protein (Fragment)	1	1	31.6	5.78	0.000243
H9J6E3	Uncharacterized protein	3	1	11.9	6.3	0.005418
H9ISK3	Uncharacterized protein	2	1	32.3	6.79	0.001323
G6CWA4	Hydroxyacyl-coenzyme A dehydrogenase	1	1	82.3	8.98	4.77E-06
H9JA67	Uncharacterized protein	4	1	10	8.92	1.17E-05
S4PCQ8	Uncharacterized protein (Fragment)	1	1	10.7	5.25	0.02039
G6DQ02	Dolichyl-diphosphooligosaccharide--protein glycosyltransferase subunit DAD1	4	1	12.6	8.18	0.000858
I4DME2	Gamma-subunit,methylmalonyl-CoA decarboxylase	4	1	14.5	8.24	0.000206
H9IV94	Uncharacterized protein	2	1	25.4	6.67	7.59E-06
H9JPK6	Uncharacterized protein	1	1	40.7	8.84	3.32E-05
A1Z076	ELAV-like protein	2	1	30.4	9.48	0.010036
I4DP00	Similar to CG1416	1	1	36.7	8.15	0.000269
S4PEG4	Alpha-1,3/1,6-mannosyltransferase ALG2 (Fragment)	2	1	26.1	8.65	1.37E-05
H9J3E2	Uncharacterized protein	3	1	30.1	7.59	0.00012
Q9NKX2	Nonclathrin coat protein gamma2-COP (Fragment)	2	1	95.1	5.55	0.019877
G6CTH3	Replication protein A3	1	1	14.6	4.55	8.31E-05
A9XTS8	cAMP response element binding protein 1	4	2	28.1	8.31	0.005285
G6DAL3	Putative Tubulin-specific chaperone	3	2	27.9	5.06	0.000612

	B					
S4PB31	Coatomer protein complex subunit zeta	1	1	20.7	5.14	0.000292
S4PKX7	Protein disulfide-isomerase	4	1	54.8	5.68	2.77E-05
G6DIN8	Uncharacterized protein	3	2	37.3	6.47	8.89E-07
G6D0D4	Uncharacterized protein	1	2	89.9	5.11	0.034132
S4PXE2	Dystroglycan	3	3	69.4	5.41	6.59E-05
G6CWH0	Putative GTP cyclohydrolase 1 type 2	3	1	29.8	5.87	0.004591
S4P781	Putative E3 ubiquitin-protein ligase UBR7	1	1	23.1	8.54	0.000159
H9J119	Uncharacterized protein	1	1	23.9	7.31	0.0083
G6CPN3	Protein disulfide-isomerase	4	1	54.7	5.53	0.010141
Q1HPY2	Transcription initiation factor TFIID subunit 12	1	1	19.6	7.05	0.021782
H9JJB7	GrpE protein homolog	2	1	24.1	7.46	2.56E-05
G6CSN6	Putative specifically Rac-associated protein	2	1	145.6	7.31	0.000288
G6DBD4	Uncharacterized protein	1	1	40.3	7.52	0.001671
H9J1Q2	Uncharacterized protein	2	1	20.2	8.97	2.00E-05
H9JYI5	Uncharacterized protein	1	1	24.7	8.79	1.03E-05
G6CMU4	Proteasome 26S non-ATPase subunit 9	1	1	21.8	6.6	0.004569
G6CP01	Uncharacterized protein	2	1	71.9	7.06	0.004183
S4P8U1	Dihydrolipoamide succinyltransferase component of 2-oxoglutarate dehydrogenase	3	1	49.7	9.19	0.036982
S4P5W3	Phospholipase A-2-activating protein (Fragment)	1	1	27.4	5.68	2.15E-05
S4PRG7	Uncharacterized protein	2	1	32.6	5.4	3.07E-05
G6D396	Uncharacterized protein	1	1	32.4	7.25	0.015762
I4DPQ6	Mitochondrial ribosomal protein S22 (Fragment)	1	1	41.2	7.01	1.21E-05
S4PD38	Serine palmitoyltransferase 1 (Fragment)	3	1	34.7	7.12	0.002565
G6CQ97	Uncharacterized protein	3	1	47.9	5.48	5.45E-08
H9JF59	Uncharacterized protein	1	1	44.2	9.86	6.96E-06
G6DJ60	Uncharacterized protein	1	1	26.6	9.45	0.001179
S4PDY1	Protein PRRC1	1	1	41.8	4.84	5.88E-05
I4DNQ4	tRNA selenocysteine associated protein secp43 (Fragment)	1	1	33.1	5.36	0.000742
G6CIX7	Uncharacterized protein	2	1	165.5	5.5	0.007906
H9ITJ2	Uncharacterized protein	2	1	47.3	5.12	0.000956
H9J2G3	Uncharacterized protein	2	1	188.6	7.91	0.000107

G6CT34	Uncharacterized protein	1	1	83.6	9.89	5.91E-05
S4PTG2	Eukaryotic initiation factor 4B protein (Fragment)	1	1	63.2	9.07	7.57E-06
H9JSK3	Uncharacterized protein	2	2	117.5	5.81	3.93E-05
H9JI79	Uncharacterized protein	1	1	50	6.73	0.000205
S4PG29	Uncharacterized protein	1	1	54.2	9.94	0.001134
H9J971	Uncharacterized protein	1	1	63.3	7.66	0.030812
G6CRF5	Uncharacterized protein	1	1	83.3	6.39	0.002194
Q8I864	Ribosomal protein S9 (Fragment)	1	2	12.8	10.58	1.33E-05
I6LGE9	Elongation factor 1-alpha (Fragment)	1590	1	38	8.27	0.001831
D3X5F2	Elongation factor 1-alpha (Fragment)	1430	1	44.8	8.6	0.000262
D4P251	Elongation factor 1 alpha (Fragment)	262	2	28	7.03	0.004816
G6D5P3	14-3-3 epsilon protein	1	1	29.6	4.81	7.73E-06
Q30CE1	Elongation factor 1-alpha (Fragment)	507	1	42.3	8.69	0.012027
Q5EPR3	Elongation factor 1-alpha (Fragment)	451	1	38.5	8.05	0.009466
G6DIF6	Histone H4	3	1	15.7	11.5	0.001416
Q2LAR4	Elongation factor 1-alpha (Fragment)	648	1	44.5	7.97	0.01285
W0ZFA9	Elongation factor 1 alpha (Fragment)	2	1	7.4	7.18	5.64E-05
G6CZR4	ATPase inhibitor-like protein	4	3	9.3	8.41	0.000749
D3X5D0	Elongation factor 1-alpha (Fragment)	23	1	43.6	7.72	0.001238
G0ZEI0	Ribosomal protein L28	1	1	15.8	11.87	1.55E-05
H9U396	Heat shock protein 90 beta	3	1	91.6	5.06	0.000105
H9JE10	Uncharacterized protein	3	1	24.1	7.05	0.000925
H9IUC3	Uncharacterized protein	3	3	173.3	8.59	0.049093
G9LPR1	UDP-glycosyltransferase UGT41D1	1	1	57.4	9.07	0.009565
D4P2D6	Elongation factor 1 alpha (Fragment)	168	1	28.1	7.03	7.03E-08
H9IXM2	Uncharacterized protein	2	1	34.2	9.7	0.014869
