# Supplementary material for: Comparative proteomics analysis of Spodoptera frugiperda cells during Autographa californica multiple nucleopolyhedrovirus infection
Source: Virol J. 2015 Aug 4;12:115. doi: 10.1186/s12985-015-0346-9 (PMC4524103; doi:10.1186/s12985-015-0346-9)
Supplement: Additional file 2: Table S2. — KEGG pathway. (PDF 187 kb) [file 12985_2015_346_MOESM2_ESM.pdf]

Map ID	Map Name	Seqs	#Seqs	URL
ko04141	Protein processing in endoplasmic reticulum	H9IZ19<br>Q3V6C6<br>Q8I6Y8<br>H9IT95<br>I4DMH6<br>I4DIQ3<br>I4DNK8<br>H9JSN0<br>I4DNH5<br>H9JX14<br>I4DMW4<br>S4PTH3<br>S4NNL1<br>H9JK13<br>H9J5V8<br>G6DBR6<br>H9J5Q7<br>G6DQ02<br>S4PKX7<br>G6CPN3<br>S4P5W3<br>H9U396	22	http://www.kegg.jp/kegg-bin/show_pathway?ko04141+K07953+K04079+K03237+K08057+K08057+K09580+K04555+K09502+K09584+K09556+K10839+K14009+K11718+K14009+K07151+K14003+K14004+K12668+K08056+K08056+K14018+K09487
ko01200	Carbon metabolism	B3TEH3<br>I4DP84<br>G0T406<br>L7R6U6<br>C9S271<br>D6BNT9<br>H9J814<br>G6CIZ7<br>G6CTA2<br>S4PW23<br>H9J2S8<br>H9JHI6<br>S4NSU9<br>H9JFB9<br>F8UEY1<br>H9IUJ6<br>H9JBL2<br>G6CY74<br>G6CTV3<br>G6CWA4<br>S4P8U1	21	http://www.kegg.jp/kegg-bin/show_pathway?ko01200+K01689+K01647+K00121+K01689+K07511+K01810+K07511+K01803+K00600+K00600+K00658+K00627+K01900+K01679+K00134+K00616+K00031+K00029+K00030+K07515+K00658

ko03010	Ribosome	Q8WQJ2<br>Q6F467<br>D1LYK5<br>Q5UAT9<br>E3UPC7<br>E3UUW3<br>S4NRF9<br>E7DZ32<br>Q95UN1<br>G6DME4<br>D1LYQ1<br>G6DAB3<br>D3X5P2<br>Q8ISR4<br>Q1HQC0<br>G6DH38<br>I4DMG3<br>I4DID4<br>Q8I864<br>G0ZEI0	20	http://www.kegg.jp/kegg-bin/show_pathway?ko03010+K02941+K02962+K02870+K02943+K02908+K02922+K02958+K02929+K02990+K02942+K02905+K02865+K02989+K02866+K02970+K02967+K02934+K02934+K02997+K02903
ko03013	RNA transport	G6DKM8<br>B7SET3<br>Q8I6Y8<br>H9CV61<br>Q1HPW4<br>A7UFP8<br>G6CMA9<br>I1E4Y7<br>G6D354<br>S4PB07<br>S4PL34<br>O44308<br>D1LAB2<br>S4NTC3<br>H9J5Q7<br>G6CSN6<br>S4PTG2<br>D3X5F2<br>Q2LAR4	19	http://www.kegg.jp/kegg-bin/show_pathway?ko03013+K12877+K03231+K03237+K03231+K03246+K03231+K13126+K03248+K12879+K14304+K14284+K03231+K03231+K03243+K14004+K05749+K03258+K03231+K03231
ko05016	Huntington's disease	Q86CZ0<br>I4DJ87<br>H9JV88<br>M9UVR1<br>G6DAS4<br>G6CKB9<br>I4DNY2<br>H9J1U7<br>H9ISH7<br>H9JBG4<br>M9T0T9<br>E9LFS8<br>I4DNC1<br>G6DL52<br>H9JA67<br>H9IUC3<br>H9IXM2	17	http://www.kegg.jp/kegg-bin/show_pathway?ko05016+K05863+K02134+K03948+K15040+K02135+K03957+K11826+K03006+K11644+K11351+K02126+K02256+K00413+K03008+K02128+K04498+K05863

ko05012	Parkinson's disease	Q86CZ0<br>I4DJ87<br>H9JV88<br>M9UVR1<br>G6DAS4<br>G6CKB9<br>I4DNK8<br>H9JBG4<br>M9T0T9<br>E9LFS8<br>I4DNC1<br>H9JA67<br>H9IXM2	13	http://www.kegg.jp/kegg-bin/show_pathway?ko05012+K05863+K02134+K03948+K15040+K02135+K03957+K04555+K11351+K02126+K02256+K00413+K02128+K05863
ko05010	Alzheimer's disease	E3UJZ8<br>I4DJ87<br>H9JV88<br>G6DAS4<br>G6CKB9<br>S4NZV0<br>H9JBG4<br>M9T0T9<br>E9LFS8<br>I4DNC1<br>F8UEY1<br>H9JA67	12	http://www.kegg.jp/kegg-bin/show_pathway?ko05010+K02183+K02134+K03948+K02135+K03957+K01408+K11351+K02126+K02256+K00413+K00134+K02128
ko00190	Oxidative phosphorylation	G6CKL7<br>I4DJ87<br>H9JV88<br>G6DAS4<br>G6CKB9<br>H9JBG4<br>M9T0T9<br>E9LFS8<br>I4DNC1<br>E2IV54<br>G6CQI5<br>H9JA67	12	http://www.kegg.jp/kegg-bin/show_pathway?ko00190+K02150+K02134+K03948+K02135+K03957+K11351+K02126+K02256+K00413+K02145+K02152+K02128
ko00010	Glycolysis / Gluconeogenesis	B3TEH3<br>G0T406<br>L7R6U6<br>D6BNT9<br>D2SNQ8<br>G6CIZ7<br>D2SNS6<br>H9JHI6<br>G6CJU9<br>G6DFF9<br>F8UEY1	11	http://www.kegg.jp/kegg-bin/show_pathway?ko00010+K01689+K00121+K01689+K01810+K00128+K01803+K00128+K00627+K00128+K15779+K00134
ko01230	Biosynthesis of amino acids	B3TEH3<br>I4DP84<br>L7R6U6<br>G6CIZ7<br>G6CTA2<br>S4PW23<br>Q1HQB5<br>F8UEY1<br>H9IUJ6<br>H9JBL2<br>G6CTV3	11	http://www.kegg.jp/kegg-bin/show_pathway?ko01230+K01689+K01647+K01689+K01803+K00600+K00600+K00789+K00134+K00616+K00031+K00030

ko03040	Spliceosome	G6DKM8 A9XZA5 H9JA60 H9JD02 G6D689 H9JR08 G6D354 Q1HPT3 S4NSX0 H9JB76	10	http://www.kegg.jp/kegg-bin/show_pathway?ko03040+K12877+K12820+K12741+K12857+K11092+K12823+K12879+K12741+K12872+K09567
ko05134	Legionellosis	H9IZ19 B7SET3 H9CV61 A7UFP8 I4DIL8 O44308 D1LAB2 D3X5F2 Q2LAR4	9	http://www.kegg.jp/kegg-bin/show_pathway?ko05134+K07953+K03231+K03231+K03231+K03233+K03231+K03231+K03231+K03231
ko05166	HTLV-I infection	Q86CZ0 Q8MYA5 H9IT95 I4DMH6 M9UVR1 S4PEX9 H9IUC3 H9IXM2	8	http://www.kegg.jp/kegg-bin/show_pathway?ko05166+K05863+K04802+K08057+K08057+K15040+K02327+K04498+K05863
ko00020	Citrate cycle (TCA cycle)	I4DP84 H9J2S8 H9JHI6 S4NSU9 H9JFB9 H9JBL2 G6CTV3 S4P8U1	8	http://www.kegg.jp/kegg-bin/show_pathway?ko00020+K01647+K00658+K00627+K01900+K01679+K00031+K00030+K00658
ko05169	Epstein-Barr virus infection	H9JCM2 G6DIQ9 D9N4J4 H9J1U7 H9JX71 G6DL52 G6D5P3 H9IUC3	8	http://www.kegg.jp/kegg-bin/show_pathway?ko05169+K03035+K15979+K15979+K03006+K03036+K03008+K06630+K04498
ko00071	Fatty acid degradation	G0T406 C9S271 H9J814 D2SNQ8 D2SNS6 G6CJU9 G6CYN2 G6CWA4	8	http://www.kegg.jp/kegg-bin/show_pathway?ko00071+K00121+K07511+K07511+K00128+K00128+K00128+K15013+K07515
ko00310	Lysine degradation	C9S271 H9J814 D2SNQ8 D2SNS6 H9J2S8 G6CJU9 G6CWA4 S4P8U1	8	http://www.kegg.jp/kegg-bin/show_pathway?ko00310+K07511+K07511+K00128+K00128+K00658+K00128+K07515+K00658

ko05203	Viral carcinogenesis	G6DIQ9 D9N4J4 H9JJE6 Q1HPQ3 G6D396 G6D5P3 G6DIF6 H9IUC3	8	http://www.kegg.jp/kegg-bin/show_pathway?ko05203+K15979+K15979+K11254+K03123+K05768+K06630+K11254+K04498
ko05168	Herpes simplex infection	Q8I6Y8 H9J1U7 R4ITA8 O77129 S4PL34 S4PG29 H9IUC3	7	http://www.kegg.jp/kegg-bin/show_pathway?ko05168+K03237+K03006+K14840+K02633+K14284+K14840+K04498
ko00040	Pentose and glucuronate interconversions	E1U7I5 D2SNQ8 U5KC00 D2SNS6 G6CJU9 G6D8L4 G9LPR1	7	http://www.kegg.jp/kegg-bin/show_pathway?ko00040+K00963+K00128+K00011+K00128+K00128+K00008+K00699
ko00230	Purine metabolism	S4PCX5 H9J1U7 H9IT11 S4PEX9 G6DFF9 S4PD63 G6DL52	7	http://www.kegg.jp/kegg-bin/show_pathway?ko00230+K10808+K03006+K01952+K02327+K15779+K01518+K03008
ko00970	Aminoacyl-tRNA biosynthesis	G6DNL4 E9KGW9 E0D4V7 H9IX66 G6CVH5 G6DFX7 G6CNG9	7	http://www.kegg.jp/kegg-bin/show_pathway?ko00970+K01875+K14163+K01890+K01868+K01876+K01870+K01883
ko00280	Valine, leucine and isoleucine degradation	C9S271 H9J814 D2SNQ8 D2SNS6 G6CJU9 G6CRM2 G6CWA4	7	http://www.kegg.jp/kegg-bin/show_pathway?ko00280+K07511+K07511+K00128+K00128+K00128+K05607+K07515
ko04144	Endocytosis	I4DNY2 S4P5Y2 S4P8C3 S4P621 Q1HQ07 G6DG28 G6CIU6	7	http://www.kegg.jp/kegg-bin/show_pathway?ko04144+K11826+K12471+K04707+K12200+K12199+K12486+K18442
ko04022	cGMP-PKG signaling pathway	E3UJZ8 Q86CZ0 M9UVR1 Q8T9W9 H9J971 H9IXM2	6	http://www.kegg.jp/kegg-bin/show_pathway?ko04022+K02183+K05863+K15040+K07376+K01540+K05863

ko05034	Alcoholism	E3UJZ8<br>Q1HPV7<br>H9IZJ3<br>H9JJE6<br>H9JY79<br>G6DIF6	6	http://www.kegg.jp/kegg-bin/show_pathway?ko05034+K02183+K11251+K06279+K11254+K11251+K11254
ko05200	Pathways in cancer	Q3V6C6<br>S4P8C3<br>H9IY28<br>H9JFB9<br>H9U396<br>H9IUC3	6	http://www.kegg.jp/kegg-bin/show_pathway?ko05200+K04079+K04707+K03870+K01679+K09487+K04498
ko04932	Non-alcoholic fatty liver disease (NAFLD)	Q8I6Y8<br>H9JV88<br>G6CKB9<br>H9JBG4<br>E9LFS8<br>I4DNC1	6	http://www.kegg.jp/kegg-bin/show_pathway?ko04932+K03237+K03948+K03957+K11351+K02256+K00413
ko00520	Amino sugar and nucleotide sugar metabolism	E1U7I5<br>D6BNT9<br>S4PN79<br>I4DNI3<br>H9JFY6<br>G6DFF9	6	http://www.kegg.jp/kegg-bin/show_pathway?ko00520+K00963+K01810+K01784+K00621+K01711+K15779
ko00240	Pyrimidine metabolism	S4PCX5<br>G6D3R7<br>H9J1U7<br>S4PEX9<br>S4PD63<br>G6DL52	6	http://www.kegg.jp/kegg-bin/show_pathway?ko00240+K10808+K13800+K03006+K02327+K01518+K03008
ko00380	Tryptophan metabolism	C9S271<br>H9J814<br>D2SNQ8<br>D2SNS6<br>G6CJU9<br>G6CWA4	6	http://www.kegg.jp/kegg-bin/show_pathway?ko00380+K07511+K07511+K00128+K00128+K00128+K07515
ko00410	beta-Alanine metabolism	C9S271<br>H9J814<br>D2SNQ8<br>D2SNS6<br>G6CJU9<br>G6CWA4	6	http://www.kegg.jp/kegg-bin/show_pathway?ko00410+K07511+K07511+K00128+K00128+K00128+K07515
ko00620	Pyruvate metabolism	D2SNQ8<br>D2SNS6<br>H9JHI6<br>G6CJU9<br>H9JFB9<br>G6CY74	6	http://www.kegg.jp/kegg-bin/show_pathway?ko00620+K00128+K00128+K00627+K00128+K01679+K00029
ko00330	Arginine and proline metabolism	E9NZS5<br>D2SNQ8<br>D2SNS6<br>G6CJU9<br>Q53EK0	5	http://www.kegg.jp/kegg-bin/show_pathway?ko00330+K00933+K00128+K00128+K00128+K00472
ko00680	Methane metabolism	B3TEH3<br>G0T406<br>L7R6U6<br>G6CTA2<br>S4PW23	5	http://www.kegg.jp/kegg-bin/show_pathway?ko00680+K01689+K00121+K01689+K00600+K00600

ko03018	RNA degradation	B3TEH3 G6CMA9 H9JMC0 L7R6U6 G6D1T2	5	http://www.kegg.jp/kegg-bin/show_pathway?ko03018+K01689+K13126+K12614+K01689+K04043
ko04066	HIF-1 signaling pathway	B3TEH3 L7R6U6 H9IY28 F8UEY1 H9IUC3	5	http://www.kegg.jp/kegg-bin/show_pathway?ko04066+K01689+K01689+K03870+K00134+K04498
ko04626	Plant-pathogen interaction	E3UJZ8 Q3V6C6 S4PEA4 H9ITJ2 H9U396	5	http://www.kegg.jp/kegg-bin/show_pathway?ko04626+K02183+K04079+K12795+K00864+K09487
ko04151	PI3K-Akt signaling pathway	Q3V6C6 G6CKL9 S4PTG2 G6D5P3 H9U396	5	http://www.kegg.jp/kegg-bin/show_pathway?ko04151+K04079+K09554+K03258+K06630+K09487
ko04612	Antigen processing and presentation	Q3V6C6 H9IT95 I4DMH6 S4PKX7 G6CPN3	5	http://www.kegg.jp/kegg-bin/show_pathway?ko04612+K04079+K08057+K08057+K08056+K08056
ko04145	Phagosome	G6CKL7 H9IT95 I4DMH6 E2IV54 G6CQI5	5	http://www.kegg.jp/kegg-bin/show_pathway?ko04145+K02150+K08057+K08057+K02145+K02152
ko04721	Synaptic vesicle cycle	G6CKL7 I4DNY2 H9JAP5 E2IV54 G6CQI5	5	http://www.kegg.jp/kegg-bin/show_pathway?ko04721+K02150+K11826+K04560+K02145+K02152
ko03050	Proteasome	H9J1E8 H9JCM2 B6VAH6 H9JX71 H9J567	5	http://www.kegg.jp/kegg-bin/show_pathway?ko03050+K02738+K03035+K02737+K03036+K02730
ko04120	Ubiquitin mediated proteolysis	H9JEB5 I4DNK8 S4P8C3 H9IY28 G6D3G4	5	http://www.kegg.jp/kegg-bin/show_pathway?ko04120+K10579+K04555+K04707+K03870+K10596
ko00561	Glycerolipid metabolism	D2SNQ8 U5KC00 D2SNS6 G6CJU9 H9ITJ2	5	http://www.kegg.jp/kegg-bin/show_pathway?ko00561+K00128+K00011+K00128+K00128+K00864
ko03008	Ribosome biogenesis in eukaryotes	G6DLM7 S4PL34 G6DB46 S4NXV8 G6CRF5	5	http://www.kegg.jp/kegg-bin/show_pathway?ko03008+K11131+K14284+K14572+K14538+K14556
ko04810	Regulation of actin cytoskeleton	K9J9U2 G6CSN6 G6D396 H9J2G3	4	http://www.kegg.jp/kegg-bin/show_pathway?ko04810+K05765+K05749+K05768+K05767

ko03015	mRNA surveillance pathway	G6DKM8 G6CMA9 S4PL34 H9JFV2	4	http://www.kegg.jp/kegg-bin/show_pathway?ko03015+K12877+K13126+K14284+K06100
ko04020	Calcium signaling pathway	E3UJZ8 Q86CZ0 M9UVR1 H9IXM2	4	http://www.kegg.jp/kegg-bin/show_pathway?ko04020+K02183+K05863+K15040+K05863
ko04915	Estrogen signaling pathway	E3UJZ8 Q3V6C6 H9IZJ3 H9U396	4	http://www.kegg.jp/kegg-bin/show_pathway?ko04915+K02183+K04079+K06279+K09487
ko03030	DNA replication	Q8MYA5 S4PEX9 S4PXM6 H9JPK6	4	http://www.kegg.jp/kegg-bin/show_pathway?ko03030+K04802+K02327+K02542+K10756
ko03420	Nucleotide excision repair	Q8MYA5 I4DMW4 S4PEX9 H9JPK6	4	http://www.kegg.jp/kegg-bin/show_pathway?ko03420+K04802+K10839+K02327+K10756
ko04110	Cell cycle	Q8MYA5 S4PXM6 G6D5P3 H9IUC3	4	http://www.kegg.jp/kegg-bin/show_pathway?ko04110+K04802+K02542+K06630+K04498
ko05322	Systemic lupus erythematosus	Q1HPV7 H9JJE6 H9JY79 G6DIF6	4	http://www.kegg.jp/kegg-bin/show_pathway?ko05322+K11251+K11254+K11251+K11254
ko00052	Galactose metabolism	E1U7I5 U5KC00 S4PN79 G6DFF9	4	http://www.kegg.jp/kegg-bin/show_pathway?ko00052+K00963+K00011+K01784+K15779
ko00500	Starch and sucrose metabolism	E1U7I5 D6BNT9 G6DFF9 G9LPR1	4	http://www.kegg.jp/kegg-bin/show_pathway?ko00500+K00963+K01810+K15779+K00699
ko05110	Vibrio cholerae infection	G6CKL7 G6CTH9 E2IV54 G6CQI5	4	http://www.kegg.jp/kegg-bin/show_pathway?ko05110+K02150+K10949+K02145+K02152
ko00625	Chloroalkane and chloroalkene degradation	G0T406 D2SNQ8 D2SNS6 G6CJU9	4	http://www.kegg.jp/kegg-bin/show_pathway?ko00625+K00121+K00128+K00128+K00128
ko00640	Propanoate metabolism	C9S271 H9J814 S4NSU9 G6CWA4	4	http://www.kegg.jp/kegg-bin/show_pathway?ko00640+K07511+K07511+K01900+K07515
ko01212	Fatty acid metabolism	C9S271 H9J814 G6CYN2 G6CWA4	4	http://www.kegg.jp/kegg-bin/show_pathway?ko01212+K07511+K07511+K15013+K07515
ko00053	Ascorbate and aldarate metabolism	D2SNQ8 D2SNS6 G6CJU9 G9LPR1	4	http://www.kegg.jp/kegg-bin/show_pathway?ko00053+K00128+K00128+K00128+K00699

ko00051	Fructose and mannose metabolism	U5KC00 G6CIZ7 H9JFY6 G6D8L4	4	http://www.kegg.jp/kegg-bin/show_pathway?ko00051+K00011+K01803+K01711+K00008
ko05205	Proteoglycans in cancer	H9JR08 S4P8C3 G6CIX7 S4PTG2	4	http://www.kegg.jp/kegg-bin/show_pathway?ko05205+K12823+K04707+K10380+K03258
ko04024	cAMP signaling pathway	E3UJZ8 H9J971 H9IUC3	3	http://www.kegg.jp/kegg-bin/show_pathway?ko04024+K02183+K01540+K04498
ko04713	Circadian entrainment	E3UJZ8 Q8T9W9 O77129	3	http://www.kegg.jp/kegg-bin/show_pathway?ko04713+K02183+K07376+K02633
ko04722	Neurotrophin signaling pathway	E3UJZ8 H9IZJ3 G6D5P3	3	http://www.kegg.jp/kegg-bin/show_pathway?ko04722+K02183+K06279+K06630
ko04910	Insulin signaling pathway	E3UJZ8 H9IZJ3 S4P8C3	3	http://www.kegg.jp/kegg-bin/show_pathway?ko04910+K02183+K06279+K04707
ko04970	Salivary secretion	E3UJZ8 Q8T9W9 H9J971	3	http://www.kegg.jp/kegg-bin/show_pathway?ko04970+K02183+K07376+K01540
ko05152	Tuberculosis	E3UJZ8 G6D1T2 H9IUC3	3	http://www.kegg.jp/kegg-bin/show_pathway?ko05152+K02183+K04043+K04498
ko04621	NOD-like receptor signaling pathway	Q3V6C6 S4PEA4 H9U396	3	http://www.kegg.jp/kegg-bin/show_pathway?ko04621+K04079+K12795+K09487
ko05215	Prostate cancer	Q3V6C6 H9U396 H9IUC3	3	http://www.kegg.jp/kegg-bin/show_pathway?ko05215+K04079+K09487+K04498
ko05164	Influenza A	Q8I6Y8 S4PL34 H9IUC3	3	http://www.kegg.jp/kegg-bin/show_pathway?ko05164+K03237+K14284+K04498
ko03430	Mismatch repair	Q8MYA5 S4PEX9 H9JPK6	3	http://www.kegg.jp/kegg-bin/show_pathway?ko03430+K04802+K02327+K10756
ko00480	Glutathione metabolism	S4PCX5 Q308N8 H9JBL2	3	http://www.kegg.jp/kegg-bin/show_pathway?ko00480+K10808+K00799+K00031
ko04966	Collecting duct acid secretion	G6CKL7 E2IV54 G6CQI5	3	http://www.kegg.jp/kegg-bin/show_pathway?ko04966+K02150+K02145+K02152
ko05120	Epithelial cell signaling in Helicobacter pylori infection	G6CKL7 E2IV54 G6CQI5	3	http://www.kegg.jp/kegg-bin/show_pathway?ko05120+K02150+K02145+K02152
ko05323	Rheumatoid arthritis	G6CKL7 E2IV54 G6CQI5	3	http://www.kegg.jp/kegg-bin/show_pathway?ko05323+K02150+K02145+K02152

ko00630	Glyoxylate and dicarboxylate metabolism	I4DP84 G6CTA2 S4PW23	3	http://www.kegg.jp/kegg-bin/show_pathway?ko00630+K01647+K00600+K00600
ko01210	2-Oxocarboxylic acid metabolism	I4DP84 H9JBL2 G6CTV3	3	http://www.kegg.jp/kegg-bin/show_pathway?ko01210+K01647+K00031+K00030
ko00830	Retinol metabolism	G0T406 H9JQH9 G9LPR1	3	http://www.kegg.jp/kegg-bin/show_pathway?ko00830+K00121+K11147+K00699
ko00980	Metabolism of xenobiotics by cytochrome P450	G0T406 Q308N8 G9LPR1	3	http://www.kegg.jp/kegg-bin/show_pathway?ko00980+K00121+K00799+K00699
ko00982	Drug metabolism - cytochrome P450	G0T406 Q308N8 G9LPR1	3	http://www.kegg.jp/kegg-bin/show_pathway?ko00982+K00121+K00799+K00699
ko05204	Chemical carcinogenesis	G0T406 Q308N8 G9LPR1	3	http://www.kegg.jp/kegg-bin/show_pathway?ko05204+K00121+K00799+K00699
ko00062	Fatty acid elongation	C9S271 H9J814 G6CWA4	3	http://www.kegg.jp/kegg-bin/show_pathway?ko00062+K07511+K07511+K07515
ko00362	Benzoate degradation	C9S271 H9J814 G6CWA4	3	http://www.kegg.jp/kegg-bin/show_pathway?ko00362+K07511+K07511+K07515
ko00627	Aminobenzoate degradation	C9S271 H9J814 G6CWA4	3	http://www.kegg.jp/kegg-bin/show_pathway?ko00627+K07511+K07511+K07515
ko00650	Butanoate metabolism	C9S271 H9J814 G6CWA4	3	http://www.kegg.jp/kegg-bin/show_pathway?ko00650+K07511+K07511+K07515
ko00930	Caprolactam degradation	C9S271 H9J814 G6CWA4	3	http://www.kegg.jp/kegg-bin/show_pathway?ko00930+K07511+K07511+K07515
ko00030	Pentose phosphate pathway	D6BNT9 G6DFF9 H9IUJ6	3	http://www.kegg.jp/kegg-bin/show_pathway?ko00030+K01810+K15779+K00616
ko00340	Histidine metabolism	D2SNQ8 D2SNS6 G6CJU9	3	http://www.kegg.jp/kegg-bin/show_pathway?ko00340+K00128+K00128+K00128
ko00903	Limonene and pinene degradation	D2SNQ8 D2SNS6 G6CJU9	3	http://www.kegg.jp/kegg-bin/show_pathway?ko00903+K00128+K00128+K00128
ko00710	Carbon fixation in photosynthetic organisms	G6CIZ7 F8UEY1 G6CY74	3	http://www.kegg.jp/kegg-bin/show_pathway?ko00710+K01803+K00134+K00029
ko00260	Glycine, serine and threonine metabolism	G6CTA2 S4PW23 S4PS76	3	http://www.kegg.jp/kegg-bin/show_pathway?ko00260+K00600+K00600+K00552

ko05206	MicroRNAs in cancer	H9IZJ3 V9VJY3 H9IUC3	3	http://www.kegg.jp/kegg-bin/show_pathway?ko05206+K06279+K11592+K04498
ko04919	Thyroid hormone signaling pathway	H9ISH7 H9J971 H9IUC3	3	http://www.kegg.jp/kegg-bin/show_pathway?ko04919+K11644+K01540+K04498
ko03022	Basal transcription factors	I4DNR5 Q1HPQ3 Q1HPY2	3	http://www.kegg.jp/kegg-bin/show_pathway?ko03022+K03139+K03123+K03126
ko05211	Renal cell carcinoma	H9IY28 H9JFB9 H9IUC3	3	http://www.kegg.jp/kegg-bin/show_pathway?ko05211+K03870+K01679+K04498
ko04260	Cardiac muscle contraction	E9LFS8 I4DNC1 H9J971	3	http://www.kegg.jp/kegg-bin/show_pathway?ko04260+K02256+K00413+K01540
ko00510	N-Glycan biosynthesis	H9J5V8 G6DQ02 S4PEG4	3	http://www.kegg.jp/kegg-bin/show_pathway?ko00510+K07151+K12668+K03843
ko00513	Various types of N-glycan biosynthesis	H9J5V8 G6DQ02 S4PEG4	3	http://www.kegg.jp/kegg-bin/show_pathway?ko00513+K07151+K12668+K03843
ko04666	Fc gamma R-mediated phagocytosis	K9J9U2 G6D396	2	http://www.kegg.jp/kegg-bin/show_pathway?ko04666+K05765+K05768
ko05133	Pertussis	K9J9U2 E3UJZ8	2	http://www.kegg.jp/kegg-bin/show_pathway?ko05133+K05765+K02183
ko04014	Ras signaling pathway	E3UJZ8 H9IZJ3	2	http://www.kegg.jp/kegg-bin/show_pathway?ko04014+K02183+K06279
ko04114	Oocyte meiosis	E3UJZ8 G6D5P3	2	http://www.kegg.jp/kegg-bin/show_pathway?ko04114+K02183+K06630
ko04261	Adrenergic signaling in cardiomyocytes	E3UJZ8 H9J971	2	http://www.kegg.jp/kegg-bin/show_pathway?ko04261+K02183+K01540
ko04720	Long-term potentiation	E3UJZ8 H9IUC3	2	http://www.kegg.jp/kegg-bin/show_pathway?ko04720+K02183+K04498
ko04740	Olfactory transduction	E3UJZ8 Q8T9W9	2	http://www.kegg.jp/kegg-bin/show_pathway?ko04740+K02183+K07376
ko04916	Melanogenesis	E3UJZ8 H9IUC3	2	http://www.kegg.jp/kegg-bin/show_pathway?ko04916+K02183+K04498
ko04971	Gastric acid secretion	E3UJZ8 H9J971	2	http://www.kegg.jp/kegg-bin/show_pathway?ko04971+K02183+K01540
ko05031	Amphetamine addiction	E3UJZ8 H9JAP5	2	http://www.kegg.jp/kegg-bin/show_pathway?ko05031+K02183+K04560
ko05214	Glioma	E3UJZ8 H9IZJ3	2	http://www.kegg.jp/kegg-bin/show_pathway?ko05214+K02183+K06279
ko03410	Base excision repair	Q8MYA5 S4PEX9	2	http://www.kegg.jp/kegg-bin/show_pathway?ko03410+K04802+K02327

ko05161	Hepatitis B	Q8MYA5 H9IUC3	2	http://www.kegg.jp/kegg-bin/show_pathway?ko05161+K04802+K04498
ko00770	Pantothenate and CoA biosynthesis	S4PGL2 H9JRQ9	2	http://www.kegg.jp/kegg-bin/show_pathway?ko00770+K01922+K09680
ko05142	Chagas disease (American trypanosomiasis)	H9IT95 I4DMH6	2	http://www.kegg.jp/kegg-bin/show_pathway?ko05142+K08057+K08057
ko04530	Tight junction	E3UKL7 H9JFV2	2	http://www.kegg.jp/kegg-bin/show_pathway?ko04530+K10352+K06100
ko00860	Porphyrin and chlorophyll metabolism	E9KGW9 G9LPR1	2	http://www.kegg.jp/kegg-bin/show_pathway?ko00860+K14163+K00699
ko04961	Endocrine and other factor-regulated calcium reabsorption	I4DNY2 H9J971	2	http://www.kegg.jp/kegg-bin/show_pathway?ko04961+K11826+K01540
ko00460	Cyanoamino acid metabolism	G6CTA2 S4PW23	2	http://www.kegg.jp/kegg-bin/show_pathway?ko00460+K00600+K00600
ko00670	One carbon pool by folate	G6CTA2 S4PW23	2	http://www.kegg.jp/kegg-bin/show_pathway?ko00670+K00600+K00600
ko00760	Nicotinate and nicotinamide metabolism	H9IV80 S4PE88	2	http://www.kegg.jp/kegg-bin/show_pathway?ko00760+K01950+K00763
ko04911	Insulin secretion	H9JAP5 H9J971	2	http://www.kegg.jp/kegg-bin/show_pathway?ko04911+K04560+K01540
ko04012	ErbB signaling pathway	H9IZJ3 S4P8C3	2	http://www.kegg.jp/kegg-bin/show_pathway?ko04012+K06279+K04707
ko05100	Bacterial invasion of epithelial cells	H9IZJ3 S4P8C3	2	http://www.kegg.jp/kegg-bin/show_pathway?ko05100+K06279+K04707
ko05220	Chronic myeloid leukemia	H9IZJ3 S4P8C3	2	http://www.kegg.jp/kegg-bin/show_pathway?ko05220+K06279+K04707
ko05202	Transcriptional misregulation in cancer	H9JR08 H9ISH7	2	http://www.kegg.jp/kegg-bin/show_pathway?ko05202+K12823+K11644
ko03020	RNA polymerase	H9J1U7 G6DL52	2	http://www.kegg.jp/kegg-bin/show_pathway?ko03020+K03006+K03008
ko04630	Jak-STAT signaling pathway	S4P8C3 H9IUC3	2	http://www.kegg.jp/kegg-bin/show_pathway?ko04630+K04707+K04498

ko00564	Glycerophospholipid metabolism	H9JQK7 H9JEI1	2	http://www.kegg.jp/kegg-bin/show_pathway?ko00564+K00967+K14676
ko04390	Hippo signaling pathway	H9J2E4 G6D5P3	2	http://www.kegg.jp/kegg-bin/show_pathway?ko04390+K16687+K06630
ko04391	Hippo signaling pathway - fly	H9J2E4 G6D5P3	2	http://www.kegg.jp/kegg-bin/show_pathway?ko04391+K16687+K06630
ko04310	Wnt signaling pathway	G6DK55 H9IUC3	2	http://www.kegg.jp/kegg-bin/show_pathway?ko04310+K04508+K04498
ko04146	Peroxisome	H9JQH9 H9JBL2	2	http://www.kegg.jp/kegg-bin/show_pathway?ko04146+K11147+K00031
ko03320	PPAR signaling pathway	G6CYN2 H9ITJ2	2	http://www.kegg.jp/kegg-bin/show_pathway?ko03320+K15013+K00864
ko04150	mTOR signaling pathway	H9ISY0 S4PTG2	2	http://www.kegg.jp/kegg-bin/show_pathway?ko04150+K08267+K03258
ko00720	Carbon fixation pathways in prokaryotes	H9JFB9 H9JBL2	2	http://www.kegg.jp/kegg-bin/show_pathway?ko00720+K01679+K00031
ko04918	Thyroid hormone synthesis	H9J971 H9U396	2	http://www.kegg.jp/kegg-bin/show_pathway?ko04918+K01540+K09487
ko04360	Axon guidance	K9J9U2	1	http://www.kegg.jp/kegg-bin/show_pathway?ko04360+K05765
ko04015	Rap1 signaling pathway	E3UJZ8	1	http://www.kegg.jp/kegg-bin/show_pathway?ko04015+K02183
ko04070	Phosphatidylinositol signaling system	E3UJZ8	1	http://www.kegg.jp/kegg-bin/show_pathway?ko04070+K02183
ko04270	Vascular smooth muscle contraction	E3UJZ8	1	http://www.kegg.jp/kegg-bin/show_pathway?ko04270+K02183
ko04728	Dopaminergic synapse	E3UJZ8	1	http://www.kegg.jp/kegg-bin/show_pathway?ko04728+K02183
ko04744	Phototransduction	E3UJZ8	1	http://www.kegg.jp/kegg-bin/show_pathway?ko04744+K02183
ko04745	Phototransduction - fly	E3UJZ8	1	http://www.kegg.jp/kegg-bin/show_pathway?ko04745+K02183
ko04750	Inflammatory mediator regulation of TRP channels	E3UJZ8	1	http://www.kegg.jp/kegg-bin/show_pathway?ko04750+K02183
ko04912	GnRH signaling pathway	E3UJZ8	1	http://www.kegg.jp/kegg-bin/show_pathway?ko04912+K02183
ko04921	Oxytocin signaling pathway	E3UJZ8	1	http://www.kegg.jp/kegg-bin/show_pathway?ko04921+K02183

ko04914	Progesterone-mediated oocyte maturation	Q3V6C6	1	http://www.kegg.jp/kegg-bin/show_pathway?ko04914+K04079
ko05160	Hepatitis C	Q8I6Y8	1	http://www.kegg.jp/kegg-bin/show_pathway?ko05160+K03237
ko05162	Measles	Q8I6Y8	1	http://www.kegg.jp/kegg-bin/show_pathway?ko05162+K03237
ko04115	p53 signaling pathway	S4PCX5	1	http://www.kegg.jp/kegg-bin/show_pathway?ko04115+K10808
ko00350	Tyrosine metabolism	G0T406	1	http://www.kegg.jp/kegg-bin/show_pathway?ko00350+K00121
ko00626	Naphthalene degradation	G0T406	1	http://www.kegg.jp/kegg-bin/show_pathway?ko00626+K00121
ko01220	Degradation of aromatic compounds	G0T406	1	http://www.kegg.jp/kegg-bin/show_pathway?ko01220+K00121
ko05146	Amoebiasis	B2X122	1	http://www.kegg.jp/kegg-bin/show_pathway?ko05146+K13963
ko00562	Inositol phosphate metabolism	G6CIZ7	1	http://www.kegg.jp/kegg-bin/show_pathway?ko00562+K01803
ko04142	Lysosome	I4DQ01	1	http://www.kegg.jp/kegg-bin/show_pathway?ko04142+K12399
ko04130	SNARE interactions in vesicular transport	H9JAP5	1	http://www.kegg.jp/kegg-bin/show_pathway?ko04130+K04560
ko04062	Chemokine signaling pathway	H9IZJ3	1	http://www.kegg.jp/kegg-bin/show_pathway?ko04062+K06279
ko04510	Focal adhesion	H9IZJ3	1	http://www.kegg.jp/kegg-bin/show_pathway?ko04510+K06279
ko04650	Natural killer cell mediated cytotoxicity	H9IZJ3	1	http://www.kegg.jp/kegg-bin/show_pathway?ko04650+K06279
ko04917	Prolactin signaling pathway	H9IZJ3	1	http://www.kegg.jp/kegg-bin/show_pathway?ko04917+K06279
ko00512	Mucin type O-Glycan biosynthesis	S4PBL3	1	http://www.kegg.jp/kegg-bin/show_pathway?ko00512+K00710
ko04080	Neuroactive ligand-receptor interaction	G6DI16	1	http://www.kegg.jp/kegg-bin/show_pathway?ko04080+K05236
ko04660	T cell receptor signaling pathway	S4P8C3	1	http://www.kegg.jp/kegg-bin/show_pathway?ko04660+K04707
ko00270	Cysteine and methionine metabolism	Q1HQB5	1	http://www.kegg.jp/kegg-bin/show_pathway?ko00270+K00789

ko04140	Regulation of autophagy	J9XNW6	1	http://www.kegg.jp/kegg-bin/show_pathway?ko04140+K08342
ko00563	Glycosylphosphatidylinositol(GPI)-anchor biosynthesis	G6CQB4	1	http://www.kegg.jp/kegg-bin/show_pathway?ko00563+K05290
ko00440	Phosphonate and phosphinate metabolism	H9JQK7	1	http://www.kegg.jp/kegg-bin/show_pathway?ko00440+K00967
ko04010	MAPK signaling pathway	G6CV32	1	http://www.kegg.jp/kegg-bin/show_pathway?ko04010+K04461
ko04340	Hedgehog signaling pathway	G6D6P8	1	http://www.kegg.jp/kegg-bin/show_pathway?ko04340+K08958
ko04540	Gap junction	Q8T9W9	1	http://www.kegg.jp/kegg-bin/show_pathway?ko04540+K07376
ko04611	Platelet activation	Q8T9W9	1	http://www.kegg.jp/kegg-bin/show_pathway?ko04611+K07376
ko04730	Long-term depression	Q8T9W9	1	http://www.kegg.jp/kegg-bin/show_pathway?ko04730+K07376
ko03440	Homologous recombination	S4PEX9	1	http://www.kegg.jp/kegg-bin/show_pathway?ko03440+K02327
ko02020	Two-component system	I4DNC1	1	http://www.kegg.jp/kegg-bin/show_pathway?ko02020+K00413
ko04710	Circadian rhythm	O77129	1	http://www.kegg.jp/kegg-bin/show_pathway?ko04710+K02633
ko04711	Circadian rhythm - fly	O77129	1	http://www.kegg.jp/kegg-bin/show_pathway?ko04711+K02633
ko03060	Protein export	H9JFU5	1	http://www.kegg.jp/kegg-bin/show_pathway?ko03060+K13431
ko00061	Fatty acid biosynthesis	G6CYN2	1	http://www.kegg.jp/kegg-bin/show_pathway?ko00061+K15013
ko04920	Adipocytokine signaling pathway	G6CYN2	1	http://www.kegg.jp/kegg-bin/show_pathway?ko04920+K15013
ko03460	Fanconi anemia pathway	G6D6E9	1	http://www.kegg.jp/kegg-bin/show_pathway?ko03460+K02350
ko04111	Cell cycle - yeast	S4PXM6	1	http://www.kegg.jp/kegg-bin/show_pathway?ko04111+K02542
ko04113	Meiosis - yeast	S4PXM6	1	http://www.kegg.jp/kegg-bin/show_pathway?ko04113+K02542
ko04622	RIG-I-like receptor signaling pathway	S4PT88	1	http://www.kegg.jp/kegg-bin/show_pathway?ko04622+K09578

ko01040	Biosynthesis of unsaturated fatty acids	G6CWA4	1	http://www.kegg.jp/kegg-bin/show_pathway?ko01040+K07515
ko04512	ECM-receptor interaction	S4PXE2	1	http://www.kegg.jp/kegg-bin/show_pathway?ko04512+K06265
ko05410	Hypertrophic cardiomyopathy (HCM)	S4PXE2	1	http://www.kegg.jp/kegg-bin/show_pathway?ko05410+K06265
ko05412	Arrhythmogenic right ventricular cardiomyopathy (ARVC)	S4PXE2	1	http://www.kegg.jp/kegg-bin/show_pathway?ko05412+K06265
ko05414	Dilated cardiomyopathy	S4PXE2	1	http://www.kegg.jp/kegg-bin/show_pathway?ko05414+K06265
ko05416	Viral myocarditis	S4PXE2	1	http://www.kegg.jp/kegg-bin/show_pathway?ko05416+K06265
ko00730	Thiamine metabolism	H9J119	1	http://www.kegg.jp/kegg-bin/show_pathway?ko00730+K04487
ko04122	Sulfur relay system	H9J119	1	http://www.kegg.jp/kegg-bin/show_pathway?ko04122+K04487
ko00600	Sphingolipid metabolism	S4PD38	1	http://www.kegg.jp/kegg-bin/show_pathway?ko00600+K00654
ko04960	Aldosterone-regulated sodium reabsorption	H9J971	1	http://www.kegg.jp/kegg-bin/show_pathway?ko04960+K01540
ko04964	Proximal tubule bicarbonate reclamation	H9J971	1	http://www.kegg.jp/kegg-bin/show_pathway?ko04964+K01540
ko04972	Pancreatic secretion	H9J971	1	http://www.kegg.jp/kegg-bin/show_pathway?ko04972+K01540
ko04973	Carbohydrate digestion and absorption	H9J971	1	http://www.kegg.jp/kegg-bin/show_pathway?ko04973+K01540
ko04974	Protein digestion and absorption	H9J971	1	http://www.kegg.jp/kegg-bin/show_pathway?ko04974+K01540
ko04976	Bile secretion	H9J971	1	http://www.kegg.jp/kegg-bin/show_pathway?ko04976+K01540
ko04978	Mineral absorption	H9J971	1	http://www.kegg.jp/kegg-bin/show_pathway?ko04978+K01540
ko04068	FoxO signaling pathway	H9IUC3	1	http://www.kegg.jp/kegg-bin/show_pathway?ko04068+K04498
ko04330	Notch signaling pathway	H9IUC3	1	http://www.kegg.jp/kegg-bin/show_pathway?ko04330+K04498

ko04350	TGF-beta signaling pathway	H9IUC3	1	http://www.kegg.jp/kegg-bin/show_pathway?ko04350+K04498
ko04520	Adherens junction	H9IUC3	1	http://www.kegg.jp/kegg-bin/show_pathway?ko04520+K04498
ko00140	Steroid hormone biosynthesis	G9LPR1	1	http://www.kegg.jp/kegg-bin/show_pathway?ko00140+K00699
ko00983	Drug metabolism - other enzymes	G9LPR1	1	http://www.kegg.jp/kegg-bin/show_pathway?ko00983+K00699
